# Myricetin slows liquid–liquid phase separation of Tau and activates ATG5-dependent autophagy to suppress Tau toxicity

**DOI:** 10.1016/j.jbc.2021.101222

**Published:** 2021-09-22

**Authors:** Bin Dai, Tao Zhong, Zhi-Xian Chen, Wang Chen, Na Zhang, Xiao-Ling Liu, Li-Qiang Wang, Jie Chen, Yi Liang

**Affiliations:** 1 Hubei Key Laboratory of Cell Homeostasis, College of Life Sciences, Wuhan University, Wuhan, China; 2 Wuhan University Shenzhen Research Institute, Shenzhen, China

**Keywords:** Tau protein, protein aggregation, protein liquid–liquid phase separation, myricetin, Alzheimer's disease, Tau toxicity, ATG, autophagy-related, ATG5, autophagy-related protein 5, FRAP, fluorescence recovery after photobleaching, IP, immunoprecipitation, LC3, microtubule-associated protein 1 light chain 3, LLPS, liquid–liquid phase separation, PI, propidium iodide, TAMRA, 5(6)-carboxy-tetramethylrhodamine N-succinimidyl ester, TEM, transmission electron microscopy

## Abstract

Intraneuronal neurofibrillary tangles composed of Tau aggregates have been widely accepted as an important pathological hallmark of Alzheimer's disease. A current therapeutic avenue for treating Alzheimer's disease is aimed at inhibiting Tau accumulation with small molecules such as natural flavonoids. Liquid–liquid phase separation (LLPS) of Tau can lead to its aggregation, and Tau aggregates can then be degraded by autophagy. However, it is unclear whether natural flavonoids modulate the formation of phase-separated Tau droplets or promote autophagy and Tau clearance. Here, using confocal microscopy and fluorescence recovery after photobleaching assays, we report that a natural antioxidant flavonoid compound myricetin slows LLPS of full-length human Tau, shifting the equilibrium phase boundary to a higher protein concentration. This natural flavonoid also significantly inhibits pathological phosphorylation and abnormal aggregation of Tau in neuronal cells and blocks mitochondrial damage and apoptosis induced by Tau aggregation. Importantly, using coimmunoprecipitation and Western blotting, we show that treatment of cells with myricetin stabilizes the interaction between Tau and autophagy-related protein 5 (ATG5) to promote clearance of phosphorylated Tau to indirectly limit its aggregation. Consistently, this natural flavonoid inhibits mTOR pathway, activates ATG5-dependent Tau autophagy, and almost completely suppresses Tau toxicity in neuronal cells. Collectively, these results demonstrate how LLPS and abnormal aggregation of Tau are inhibited by natural flavonoids, bridging the gap between Tau LLPS and aggregation in neuronal cells, and also establish that myricetin could act as an ATG5-dependent autophagic activator to ameliorate the pathogenesis of Alzheimer's disease.

The major physiological function of Tau, a microtubule-associated protein, is the stabilization of neuronal microtubules through the microtubule-binding region of Tau protein (1, 2, 3, 4, 5, 6). Under pathological conditions, Tau detaches from microtubules and folds into amyloid fibrils, which leads to several neurodegenerative diseases known as tauopathies, including Alzheimer's disease (1, 7, 8). The abnormal aggregates of Tau are often severely modified, most commonly through hyperphosphorylation, and regulated by several factors (8, 9, 10, 11). A current therapeutic strategy for treating tauopathies is aimed at inhibiting Tau accumulation with small molecules such as natural flavonoids.

Cells have evolved liquid–liquid phase separation (LLPS) of proteins with natively unfolded and/or low-complexity domains for liquid-phase condensation that generates membrane-less organelles to perform key functions such as stress granule formation (12, 13, 14, 15, 16, 17). Because of its natively unfolded structure and inhomogeneous charge distribution, Tau undergoes LLPS, which leads to the aggregation of the protein (12, 13, 14, 15, 16, 17, 18, 19, 20, 21). Tau liquid-phase condensation is modulated by disease-associated modifications and other factors (12, 13, 14, 15, 16, 17, 18, 19, 20, 21). However, it is unclear whether natural flavonoids modulate the formation of phase-separated Tau droplets.

Although efforts have been dedicated to unravel the pathogenic mechanisms of neurodegenerative diseases, there are no effective therapeutic approaches to prevent or cure such diseases so far (22, 23, 24, 25). Several organic molecules have shown their ability to inhibit fibril formation *in vitro* and thus represent an increasing list of potential antiamyloid compounds (22, 23, 24, 25, 26). Polyphenols including myricetin have been proposed to be active in many pathways mostly centered on their antioxidative properties (27, 28, 29). Autophagy is a conservative catabolic pathway through lysosomal degradation of intracellular components (30, 31, 32) and appears to degrade aggregate-prone proteins that link to a range of neurodegenerative diseases (33, 34, 35). At least 35 genes coding autophagy-related (ATG) proteins, including autophagy-related protein 5 (ATG5) and microtubule-associated protein 1 light chain 3 (LC3), were identified, in which LLPS plays an important role in autophagosome formation (36, 37, 38, 39, 40). ATG5, having a molecular weight of 32 kDa, is an E3 ubiquitin ligase essential for autophagosome elongation and consists of two ubiquitin-fold domains 1 and 2 linked by a helix-rich domain (36, 37, 39). Currently, most aggregate-prone proteins have been shown to be autophagy substrates (41, 42), and Tau aggregates can be degraded by autophagy. However, it is unclear whether natural flavonoids promote autophagy and Tau clearance.

In order to bridge the gap between Tau LLPS and aggregation in neuronal cells, we used myricetin, a natural antioxidant flavonoid compound, to link Tau LLPS, Tau aggregation, and Tau autophagy. Our results indicate that myricetin slowed LLPS of full-length human Tau, significantly decreased pathological phosphorylation of Tau, and blocked mitochondrial damage induced by Tau aggregation. We chose full-length human Tau because studies with the full-length protein would be far more significant (12, 14, 15, 16, 20, 21). What is more, we demonstrated that myricetin cleared phosphorylated Tau and ameliorated Tau toxicity *via* activating ATG5-dependent Tau autophagy in neuronal cells. Our results provide direct evidence that myricetin could become a potential drug candidate for Alzheimer's disease and other tauopathies.

## Results

### Myricetin slows down LLPS of full-length Tau protein

**Figure 1:**
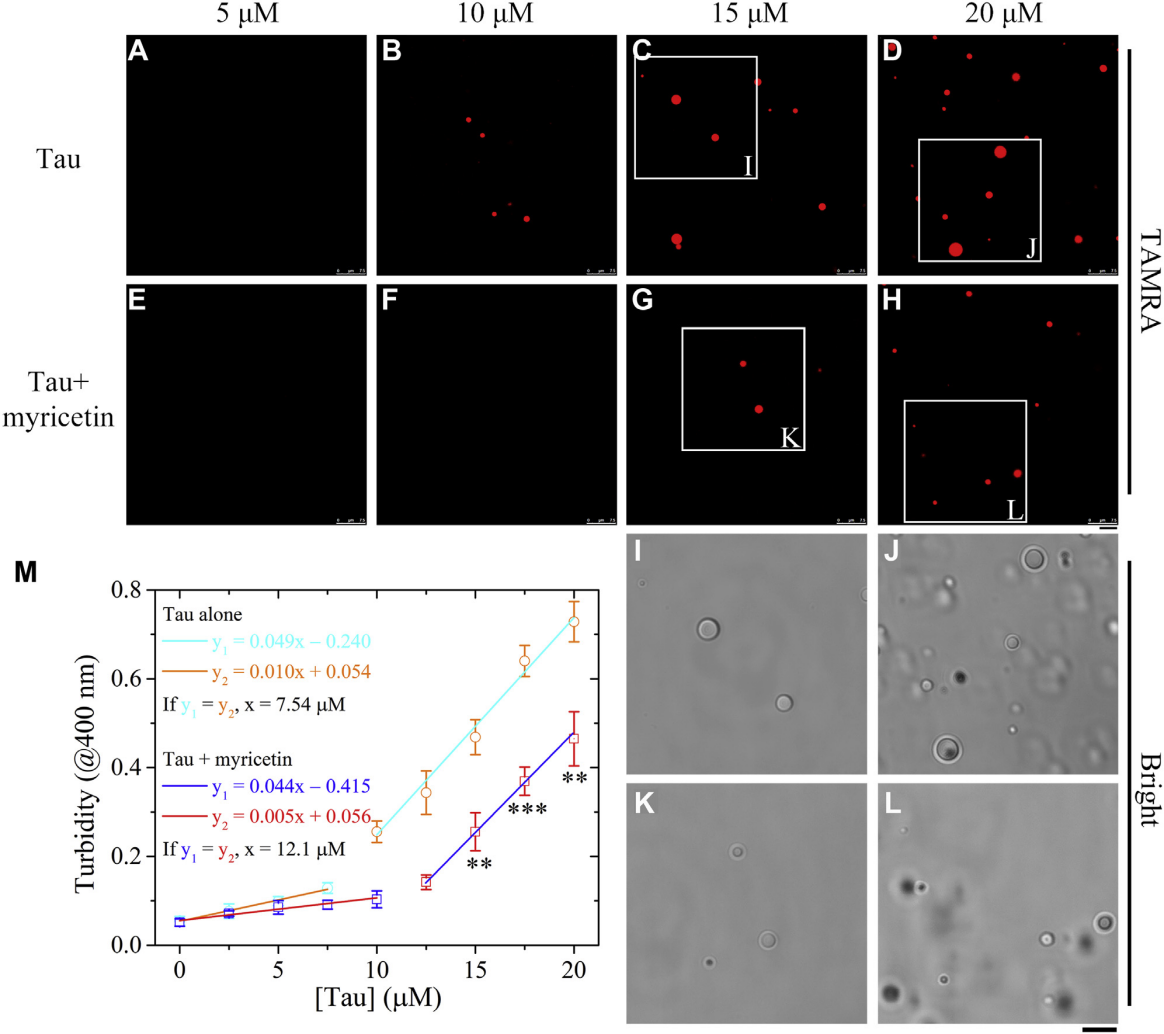
**Myricetin slows down liquid–liquid phase separation of full-length Tau protein.** 5 (*A* and *E*), 10 (*B* and *F*), 15 (*C*, *G*, *I*, and *K*), or 20 (*D*, *H*, *J*, and *L*) μM full-length human Tau was labeled by TAMRA (*red fluorescence*) and incubated with 10 mM HEPES buffer (pH 7.4) containing 10% (w/v) polyethylene glycol (PEG) 4000 and 2 mM β-mercaptoethanol or incubated with the same buffer further containing 10 μM myricetin at 25 °C to induce LLPS for 5 min. *A*–*H*, liquid droplets of Tau were observed by confocal microscopy, with excitation at 546 nm. The enlarged regions *I*, *J*, *K*, and *L* show 4-fold enlarged images from *C*, *D*, *G*, and *H*, respectively, and display the brightfield images for Tau LLPS. The scale bars represent 5 μm (*A*−*L*). *M*, the dependence of turbidity changes for LLPS of full-length Tau in the presence of 10 μM myricetin on the concentration of Tau protein ([Tau]) was expressed as mean ± S.D. (with error bars) of values obtained in three independent experiments. Representative calculation based on turbidity measurements to determine saturation concentration in the absence of myricetin (*open circle*) or in the presence of 10 μM myricetin (*open square*). The *orange line* is drawn through data points indicating the absence of LLPS, while the *cyan line* is drawn through data points in which robust LLPS occurs in the absence of myricetin. The *red line* is drawn through data points indicating the absence of LLPS, while the *blue line* is drawn through data points in which robust LLPS occurs in the presence of 10 μM myricetin. The concentration of protein at which these two lines intersect is an estimation of the saturation concentration. Saturation concentration was 7.54 μM in the absence of myricetin and 12.1 μM in the presence of 10 μM myricetin. 10 μM myricetin significantly decreased the turbidity of liquid droplets formed by 15 μM full-length Tau (0.256 ± 0.043 for Tau + myricetin *versus* 0.469 ± 0.039 for Tau alone), 17.5 μM full-length Tau (0.369 ± 0.032 for Tau + myricetin *versus* 0.640 ± 0.035 for Tau alone), or 20 μM full-length Tau (0.465 ± 0.061 for Tau + myricetin *versus* 0.729 ± 0.045 for Tau alone). The turbidity of Tau condensates was measured at 400 nm and 25 °C, and turbidity changes for Tau LLPS in the absence of myricetin were used as controls. Statistical analyses were performed using the Student's *t* test. Values of *p* < 0.05 indicate statistically significant differences. The following notation is used throughout: ∗*p* < 0.05; ∗∗*p* < 0.01; and ∗∗∗*p* < 0.001 relative to controls.

Studies with full-length Tau would be much more meaningful and informative, as the latter protein undergoes LLPS in the absence of any polyanions and at physiologically relevant concentrations (12, 14, 15, 16, 20, 21). In this article, we observed LLPS of full-length human Tau in HEPES buffer upon the addition of a crowding agent, polyethylene glycol (PEG) 4000, to mimic cellular crowded environments accurately, and studied the influence of myricetin on the formation of phase-separated Tau droplets in the presence of a reducing agent β-mercaptoethanol using confocal microscopy (Fig. 1). In total, 5 (Fig. 1, *A* and *E*), 10 (Fig. 1, *B* and *F*), 15 (Fig. 1, *C*, *G*, *I*, and *K*) or 20 (Fig. 1, *D*, *H*, *J*, and *L*) μM full-length Tau protein was labeled by 5(6)-carboxy-tetramethylrhodamine N-succinimidyl ester (TAMRA, red fluorescence) and incubated with 10 mM HEPES buffer (pH 7.4) containing 10% (w/v) PEG 4000 and 2 mM β-mercaptoethanol or incubated with the same buffer further containing 10 μM myricetin at 25 °C to induce LLPS for 5 min. Liquid droplets of Tau were observed by confocal microscopy, with excitation at 546 nm (Fig. 1, *A–H*). The enlarged regions show the brightfield images for Tau LLPS (Fig. 1, *I*, *J*, *K*, and *L*). In total, 10 μM full-length Tau did form a few liquid droplets, and 15 or 20 μM full-length Tau formed abundant liquid droplets in HEPES buffer containing 10% PEG 4000, the reducing agent, and no myricetin (Fig. 1, *B–D*). However, 10 μM full-length Tau did not form any liquid droplets, 15 μM full-length Tau did produce a few liquid droplets, and 20 μM full-length Tau formed abundant liquid droplets in the same buffer coincubated with 10 μM myricetin (Fig. 1, *F–H*). A generally accepted measure of protein propensity for LLPS is the saturation concentration (16, 19, 43). To assess the effect of myricetin on Tau propensity for LLPS, we determined saturation concentrations of full-length Tau without or with the compound by measuring the turbidity of Tau condensates at 400 nm (in the presence of 10% PEG 4000 and 2 mM β-mercaptoethanol) as a function of the concentration of Tau protein (Fig. 1*M*). These data clearly demonstrate that the saturation concentration, the concentration above which full-length Tau starts to form liquid droplets, was 7.54 μM in the absence of myricetin and 12.1 μM in the presence of 10 μM myricetin (Fig. 1*M*). We found that under reducing conditions, 10 μM myricetin did significantly decrease the turbidity of liquid droplets formed by 15 μM (*p* = 0.0032), 17.5 μM (*p* = 0.00059), or 20 μM (*p* = 0.0039) full-length Tau (Fig. 1*M*). Together, the data showed that myricetin slows down LLPS of full-length Tau protein, shifting the equilibrium phase boundary to a higher protein concentration.

**Figure 2:**
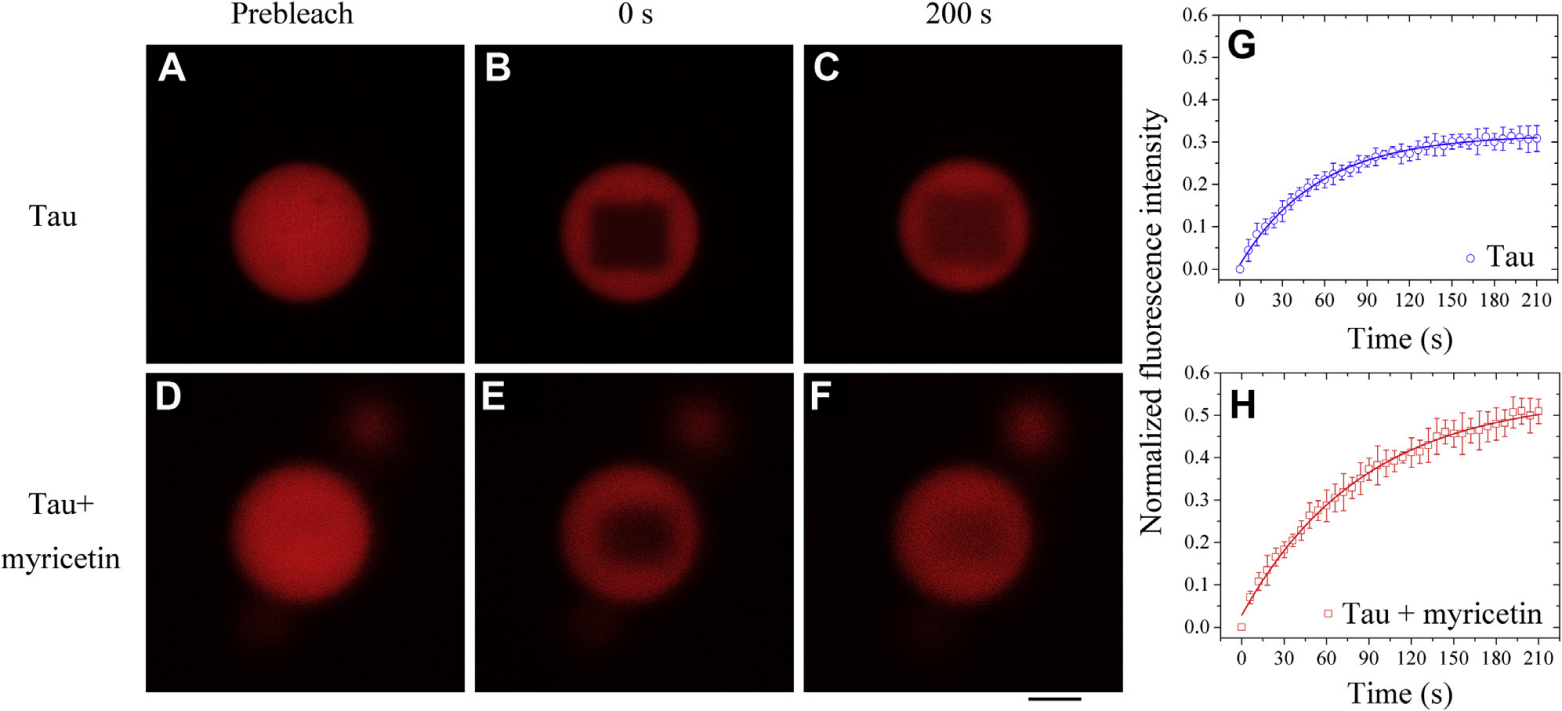
**Myricetin enhances fluorescence recovery and modulates liquid–liquid phase separation of full-length Tau protein.***A*−*F*, FRAP analysis on the selected liquid droplets of 20 μM full-length human Tau labeled by TAMRA (*red fluorescence*) before (prebleach, *A* and *D*), during (0 s, *B* and *E*), and after photobleaching (200 s, *C* and *F*). The internal photobleaching is marked by a *black square*. Full-length Tau was incubated with 10 mM HEPES buffer (pH 7.4) containing 10% (w/v) PEG 4000 and 2 mM β-mercaptoethanol (*A*−*C*) or incubated with the same buffer further containing 10 μM myricetin (*D*−*F*) at 25 °C to induce LLPS for 30 min, and liquid droplets were observed by confocal microscopy, with excitation at 546 nm. The scale bars represent 1 μm. *G* and *H*, normalized kinetics of fluorescence recovery data of Tau (*open blue circle*) and Tau + myricetin (*open red square*) obtained from FRAP intensity. The normalized fluorescence intensity is expressed as the mean ± SD of the values obtained in three independent experiments. The *solid blue* (*G*) or *red* (*H*) *lines* show the best single exponential fit for the fluorescence intensity–time curves. FRAP of phase-separated droplets of full-length Tau incubated with HEPES (*G*) or incubated with myricetin (*H*) revealed a fluorescence recovery rate of (1.66 ± 0.22) × 10^−2^ s^−1^ or (1.13 ± 0.08) × 10^−2^ s^−1^ with a (30.7 ± 1.0)% or (52.4 ± 1.4)% fluorescence recovery within 210 s. All FRAP experiments were repeated three times and the results were reproducible.

We then investigated and evaluated the dynamics of phase-separated droplets of full-length human Tau in the presence of 10% PEG 4000 and 2 mM β-mercaptoethanol by fluorescence recovery after photobleaching (FRAP) (Fig. 2, *A–H*). After photobleaching of liquid droplets of 20 μM full-length Tau protein without myricetin, a (30.7 ± 1.0)% recovery of the Tau fluorescence was observed with a fluorescence recovery rate of (1.66 ± 0.22) × 10^−2^ s^−1^ within 210 s (Fig. 2, *A–C*, and *G*). After photobleaching of liquid droplets of 20 μM full-length Tau formed in the same buffer with 10 μM myricetin, however, a (52.4 ± 1.4)% recovery of the Tau fluorescence was observed with a fluorescence recovery rate of (1.13 ± 0.08) × 10^−2^ s^−1^ within 210 s (Fig. 2, *D–F*, and *H*). The above experiments help drive the narrative that myricetin enhances fluorescence recovery and modulates LLPS of full-length Tau protein under reducing conditions.

### Myricetin slows down the formation of stress granules containing Tau in cells

**Figure 3:**
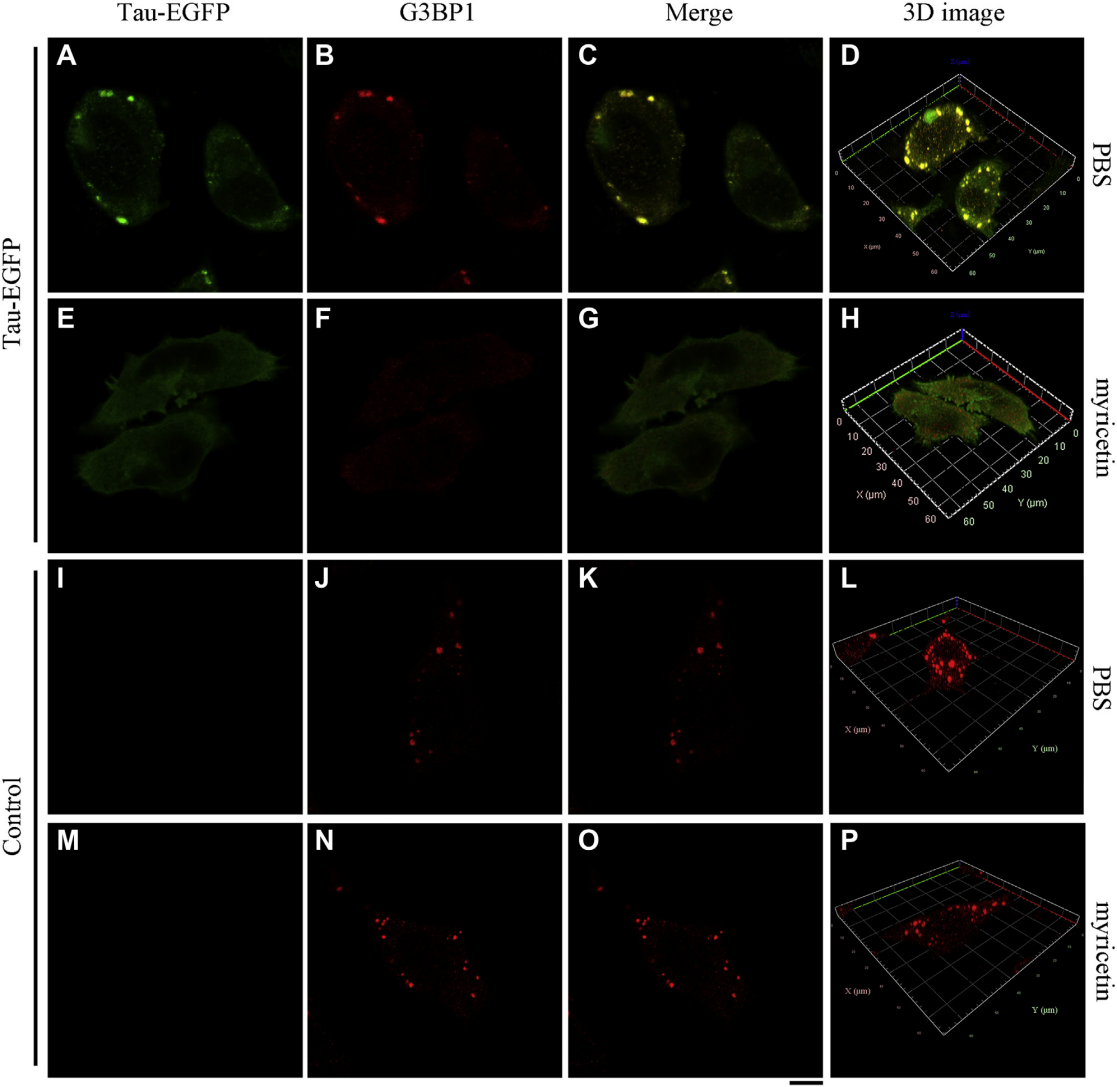
**Myricetin slows down the formation of stress granules containing Tau in cells.** SH-SY5Y cells overexpressing Tau-EGFP (*green*) with endogenous G3BP1 were incubated with 10 μM Congo red for 2 days, cultured without myricetin (*A*−*D*) or with 10 μM myricetin (*E*−*H*) for 2 days, then incubated 500 μM sodium arsenite for 45 min, fixed, permeabilized, immunostained with the anti-G3BP1 (*red*) antibody, and observed by Zeiss LSM 880 with Airyscan confocal microscopy. The confocal microscopy (Zeiss) images (*B*, *C*, *F*, and *G*) show that myricetin significantly inhibits the formation of stress granules containing Tau in cells. 3D images (*D* and *H*) display that myricetin significantly reduces colocalization of Tau and stress granules in SH-SY5Y cells. SH-SY5Y cells with empty vector and endogenous G3BP1 were incubated with 10 μM Congo red for 2 days, cultured without myricetin (*I*−*L*) or with 10 μM myricetin (*M*−*P*) for 2 days, then incubated 500 μM sodium arsenite for 45 min, fixed, permeabilized, immunostained with the anti-G3BP1 (*red*) antibody, and observed by Zeiss LSM 880 with Airyscan confocal microscopy. The confocal microscopy (Zeiss) images (*J* and *N*) and 3D images (*L* and *P*) show that myricetin has no impact on stress granule formation in SH-SY5Y cells with empty vector. The scale bars represent 10 μm.

Cells use LLPS to reversibly assemble stress granules and other membrane-less organelles (12, 13, 14, 15, 16, 17). We have shown that myricetin acts as a mild inhibitor for the formation of phase-separated Tau droplets *via* increasing the saturation concentration for Tau LLPS. We wanted to know whether myricetin could suppress stress granule formation of Tau in neuronal cells. G3BP1, a molecular switch, triggers phase separation to assemble stress granules (44). Congo red, an anionic dye that is able to overcome the energy barrier for Tau aggregation and penetrate the cell membrane (11, 17, 45, 46), was used as a modulator for Tau LLPS in cells. SH-SY5Y cells overexpressing Tau-EGFP (or FLAG-tagged Tau) with endogenous G3BP1 were incubated with 10 μM Congo red for 2 days, cultured without myricetin (Fig. 3, *A–D*) (or Fig. S1, *A-D*) or with 10 μM myricetin (Fig. 3, *E–H*) (or Fig. S1, *E-H*) for 2 days, then incubated 500 μM sodium arsenite for 45 min, fixed, permeabilized, immunostained with the anti-G3BP1 (red) antibody, and observed by Zeiss LSM 880 with Airyscan confocal microscopy or immunostained with mouse anti-FLAG antibody (green) and the anti-G3BP1 (red) antibody and observed by confocal microscopy. Under stress conditions, the colocalization of Tau and G3BP1 was clearly observed in SH-SY5Y cells when treated without myricetin (Fig. 3, *A–D*, and Fig. S1, *A–D*). The abundant yellow dots observed in 3D images represented the colocalization of Tau-GFP and G3BP1 (Figs. 3*D* and S1*D*); however, yellow dots were not observed when cells were treated with myricetin (Figs. 3*H* and S1*H*), indicating that myricetin significantly reduces the colocalization of Tau and stress granules in two cells (Fig. 3, *A–H*) and in a much larger number of cells (Fig. S1, *A–H*). Furthermore, these experiments were repeated under stress conditions (*i.e.*, arsenite) without overexpression of Tau to test if myricetin inhibits stress granule formation independent of the protein (Fig. 3, *I–P*, and Fig. S1, *I–P*). SH-SY5Y cells without overexpressing Tau-EGFP and FLAG-tagged Tau but with endogenous G3BP1 were treated and observed in the same way as above. Comparison of untreated and myricetin-treated cells under stress conditions without overexpression of Tau showed few changes (Fig. 3, *I–P*, and Fig. S1, *I–P*), indicating that myricetin inhibits stress granule formation dependent of Tau. We then evaluated and quantified such a much larger number of cells (Fig. S2). Quantification of images of G3BP1-positive stress granules performed on three biological replicates shows that myricetin significantly reduces the number of stress granules containing Tau in ∼70 cells stably overexpressing Tau (1.18 ± 1.01 stress granules per cell for Tau + myricetin *versus* 2.69 ± 1.40 stress granules per cell for Tau alone, *p* = 7.3 × 10^−11^; Fig. S2, *A* and *B*) but has no impact on the number of stress granules in ∼70 cells with empty vector (2.60 ± 1.43 stress granules per cell for control + myricetin *versus* 2.71 ± 1.44 stress granules per cell for control, *p* = 0.66; Fig. S2, *C* and *D*). Together, these results demonstrate that myricetin slows down the formation of stress granules containing Tau in neuronal cells.

### Myricetin significantly inhibits pathological phosphorylation and aggregation of Tau protein in cells

**Figure 4:**
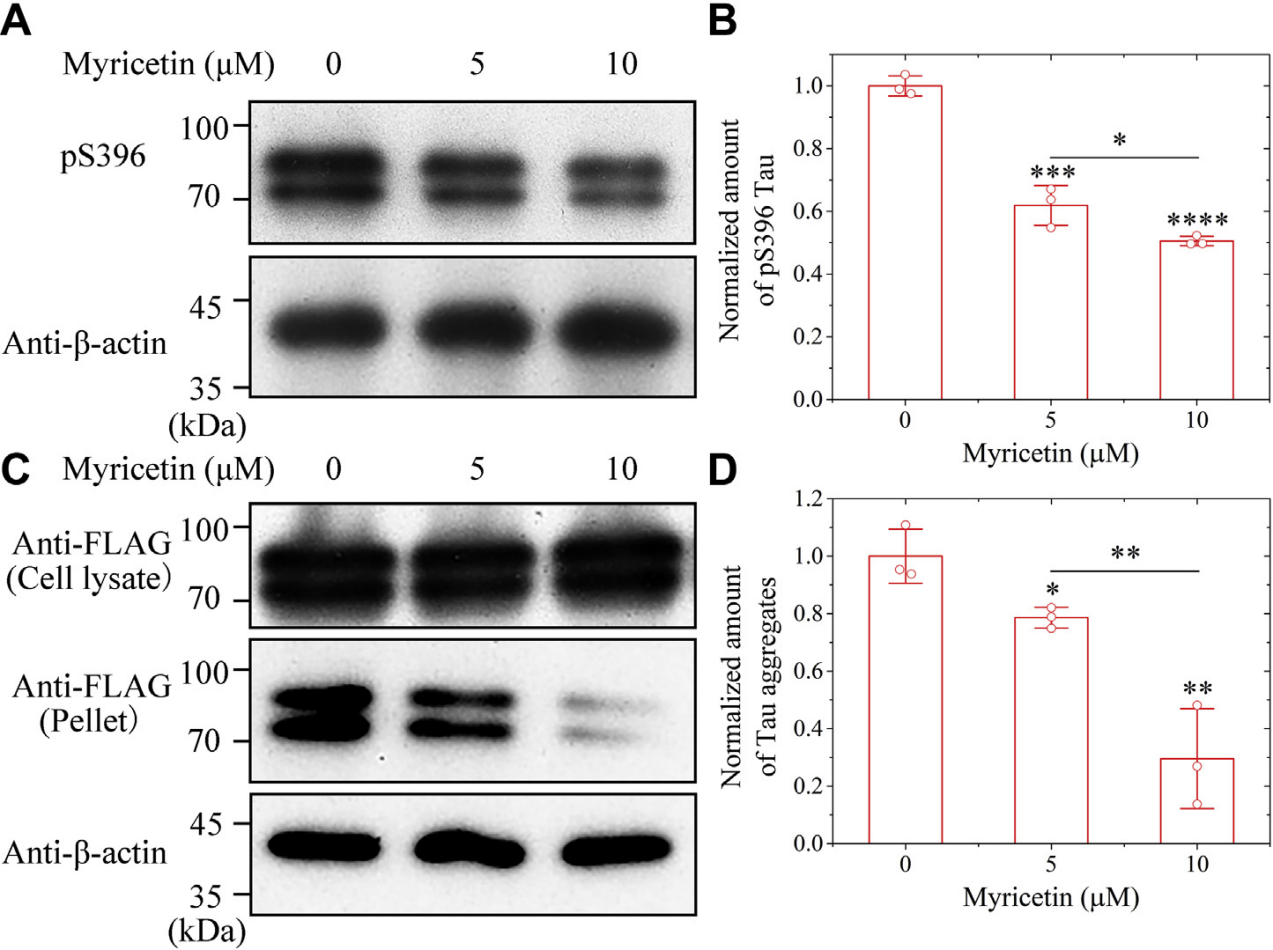
**Myricetin significantly inhibits pathological phosphorylation and aggregation of Tau protein in cells.** SH-SY5Y neuroblastoma cells stably overexpressing full-length human Tau were incubated with 10 μM Congo red for 2 days and then cultured with 0 or 10 μM myricetin for 2 days. *A*, the cell lysates from the above cells were probed by the anti-pS396 antibody and anti-β-actin antibody, respectively. *B*, the normalized amount of pS396 Tau in SH-SY5Y cells overexpressing Tau (*open red circles* shown in scatter plots) was determined as a ratio of the density of pS396 Tau bands over the density of β-actin band in cell lysates and expressed as the mean ± S.D. (with error bars) of values obtained in three independent experiments. *C*, the sarkosyl-insoluble pellets from the above cells were probed using anti-FLAG antibody, and the corresponding cell lysates were probed using anti-FLAG antibody and anti-β-actin antibody, respectively. All blots also show the position of the molecular-weight markers. *D*, the normalized amount of insoluble Tau aggregates in SH-SY5Y cells overexpressing Tau (*open red circles* shown in scatter plots) was determined as a ratio of the density of insoluble Tau aggregate bands over that of the total Tau bands in cell lysates and expressed as mean ± S.D. (with error bars) of values obtained in three independent experiments. The normalized amount of Tau aggregates in the detergent-insoluble pellets treated with an increased concentration of myricetin was significantly lower than that in the control pellets (0.786 ± 0.036 for Tau +5 μM myricetin and 0.296 ± 0.174 for Tau +10 μM myricetin *versus* 1.000 ± 0.094 for Tau alone). SH-SY5Y cells overexpressing Tau treated without myricetin were used as a control. Statistical analyses were performed using the Student's *t* test. Values of *p* < 0.05 indicate statistically significant differences. The following notation is used throughout: ∗*p* < 0.05; ∗∗*p* < 0.01; ∗∗∗*p* < 0.001; and ∗∗∗∗*p* < 0.0001 relative to control.

Cells also use LLPS to irreversibly assemble phosphorylated Tau aggregates (12, 13, 14, 15, 16, 17, 18, 19, 20, 21). We have shown that myricetin acts as a strong inhibitor for the formation of stress granules containing Tau in neuronal cells. We wanted to know whether myricetin could suppress the aggregation of phosphorylated Tau protein in neuronal cells. Congo red was used as an inducer for Tau aggregation in cells (11, 17, 45, 46). SH-SY5Y cells stably expressing full-length human Tau formed phosphorylated Tau aggregates when incubated with 10 μM Congo red for 2 days and then treated with 0, 5, or 10 μM myricetin for 2 days (Fig. 4, *A* and *C*, and Fig. S3). The cells treated with 10 μM myricetin produced much fewer phosphorylated Tau aggregates than those treated without myricetin (Fig. 4, *B* and *D*). To gain a quantitative understanding of how myricetin modulates pathological phosphorylation and aggregation of Tau in cells, we detected phosphorylated Tau and β-actin in cell lysates by using the anti-pS396 antibody and anti-β-actin antibody, respectively (Figs. 4*A* and S3, *A–C*). The normalized amount of pS396 Tau in the cell lysates treated with an increased concentration of myricetin was significantly lower than that in the control cell lysates (0.618 ± 0.063 for Tau +5 μM myricetin and 0.505 ± 0.015 for Tau +10 μM myricetin *versus* 1.000 ± 0.032 for Tau alone, *p* = 0.00075 and 0.000017, respectively) (Fig. 4*B*). We then detected insoluble Tau aggregates in the sarkosyl-insoluble ultracentrifugation pellets using anti-FLAG antibody (Figs. 4*C* and S3, *D–F*). We found that ∼10% of total Tau ended up in the pellet when the cells were treated without myricetin. There is a decrease in pellet size when myricetin is employed; however, the amount of Tau in the cell lysates does not seem to be affected (Figs. 4*C* and S3, *D–F*). The normalized amount of Tau aggregates in the detergent-insoluble pellets treated with an increased concentration of myricetin was also significantly lower than that in the control pellets (*p* = 0.020 and 0.003) (Fig. 4*D*). Our immunoblotting data demonstrated that myricetin ameliorated pathological phosphorylation and aggregation of Tau in SH-SY5Y cells, depending on the concentration of myricetin, but had no impact on the expression level of Tau. Furthermore, we performed control experiments that monitor Tau in the absence of Congo red. In the absence of Congo red, we did not observe Tau aggregates in the detergent-insoluble pellets when the cells were treated with 10 μM myricetin or treated without myricetin (Fig. S4). Together, these results demonstrate that myricetin significantly inhibits pathological phosphorylation and aggregation of Tau in neuronal cells.

### Myricetin inhibits mTOR pathway and activates ATG5-dependent Tau autophagy

**Figure 5:**
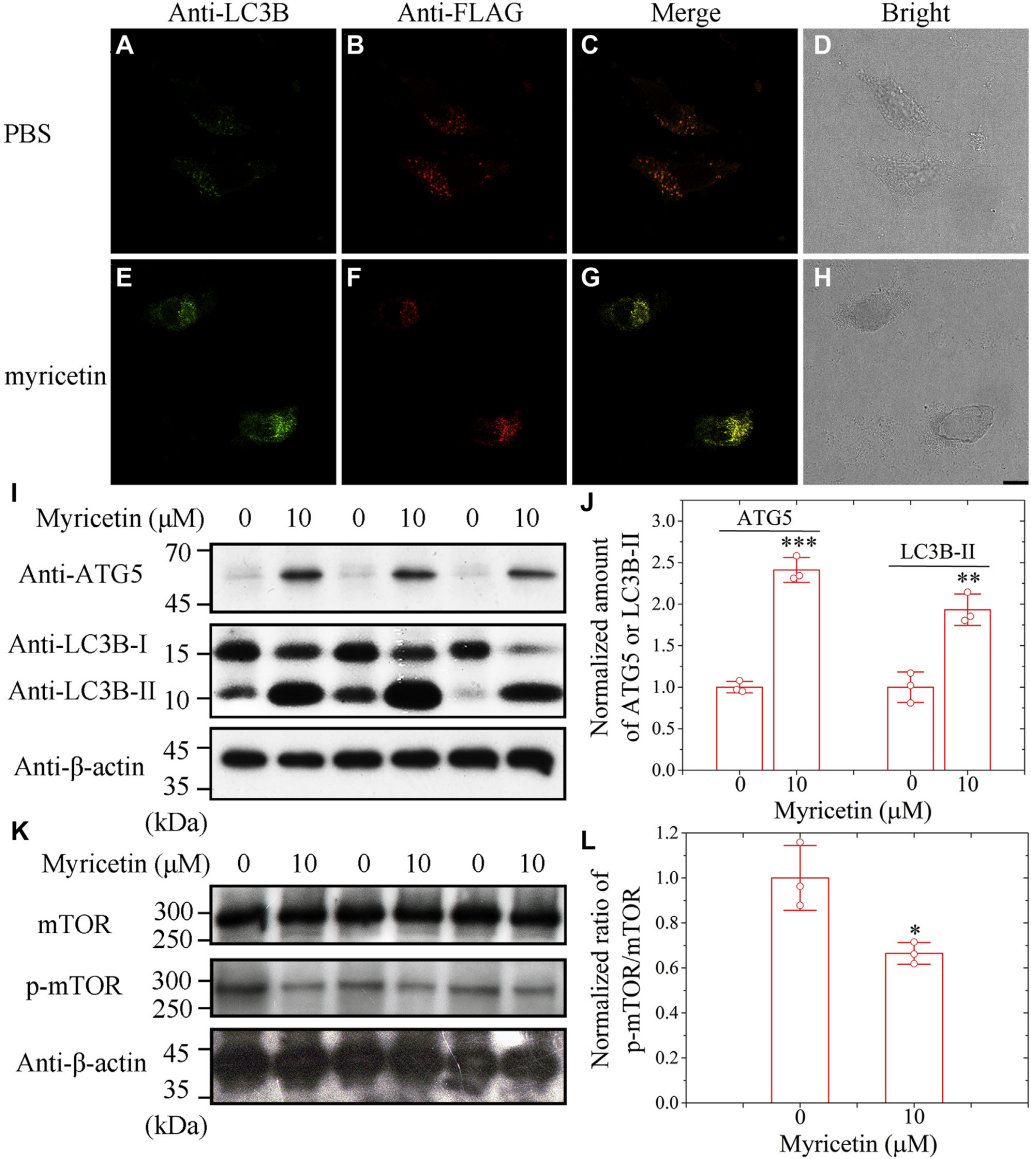
**Myricetin inhibits mTOR pathway and activates ATG5-dependent Tau autophagy.***A*−*H*, SH-SY5Y cells stably overexpressing full-length human Tau were incubated with 10 μM Congo red for 2 days and then incubated with 0 μM myricetin (*A*–*D*) or 10 μM myricetin (*E*−*H*) for 2 days. SH-SY5Y cells were coimmunostained with anti-LC3B antibody (*green*) and anti-FLAG antibody (*red*) and visualized by confocal microscopy. The scale bar represents 10 μm. *I*, the cell lysates from the above cells were probed by the anti-ATG5 antibody, anti-LC3B antibody, and anti-β-actin antibody, respectively. *J*, the normalized amount of ATG5 and LC3B-II in SH-SY5Y cells stably overexpressing Tau (*open red circles* shown in scatter plots) was determined as a ratio of the density of ATG5 or LC3B-II bands over the density of β-actin band in cell lysates and expressed as the mean ± S.D. (with error bars) of values obtained in three independent experiments. The normalized amounts of ATG5 and LC3B-II in the cell lysates treated with 10 μM myricetin were significantly higher than those in the control cell lysates treated without myricetin (2.41 ± 0.15 for ATG5 + myricetin *versus* 1.00 ± 0.07 for ATG5 alone; and 1.93 ± 0.19 for LC3B-II + myricetin *versus* 1.00 ± 0.18 for LC3B-II alone). *K*, the cell lysates from the above cells were probed by the anti-mTOR antibody, anti-p-mTOR antibody, and anti-β-actin antibody, respectively. *L*, the normalized amount of mTOR/p-mTOR in SH-SY5Y cells overexpressing Tau (*open red circles* shown in scatter plots) was determined as a ratio of the density of mTOR bands over the density of p-mTOR band in cell lysates and expressed as the mean ± S.D. (with error bars) of values obtained in three independent experiments. The normalized amount of p-mTOR/mTOR in the cell lysates treated with 10 μM myricetin was significantly lower than that in the control cell lysates (0.67 ± 0.05 for p-mTOR/mTOR + myricetin *versus* 1.00 ± 0.14 for p-mTOR/mTOR alone). SH-SY5Y cells overexpressing Tau treated without myricetin were used as controls (*J* and *L*). Statistical analyses were performed using the Student's *t* test. Values of *p* < 0.05 indicate statistically significant differences. The following notation is used throughout: ∗*p* < 0.05; ∗∗*p* < 0.01; and ∗∗∗*p* < 0.001 relative to control.

Next, we wonder whether the autophagy/lysosomal degradation pathway could participate in the reduction of pathological phosphorylation and aggregation of Tau. The formation of autophagosomes can be conveniently assayed in cells by detecting the subcellular distribution of LC3 (38). In the presence of myricetin, Tau did form abundant LC3B-positive puncta (green dots, Fig. 5*E*; and yellow dots, Fig. 5*G*) in cells; however, Tau produced much fewer LC3B-positive puncta when incubated without myricetin (green dots, Fig. 5*A*; and orange red dots, Fig. 5*C*), as detected by immunofluorescence using anti-LC3B antibody (red) and anti-FLAG antibody (green). Interestingly, we observed the colocalization of Tau and LC3B is strongly enhanced by myricetin (Fig. 5, *A–H*), suggesting that treatment of cells with myricetin stabilizes the interaction between Tau and LC3B. To gain a quantitative understanding of how myricetin activates Tau autophagy in cells, we detected three autophagy-related proteins ATG5, LC3B, and p-mTOR from the above cells using anti-ATG5, anti-LC3B, and anti-p-mTOR antibodies, respectively (Figs. 5, *I* and *K*, and S5, *A* and *B*). The normalized amounts of ATG5 and LC3B-II in the cell lysates treated with 10 μM myricetin were significantly higher than those in the control cell lysates treated without myricetin (*p* = 0.00012 and 0.003) (Fig. 5, *I* and *J*). The normalized amount of p-mTOR/mTOR in the cell lysates treated with 10 μM myricetin was significantly lower than that in the control cell lysates (*p* = 0.019) (Fig. 5, *K* and *L*). Thus, myricetin inhibits mTOR pathway, enhances the levels of ATG5 and LC3B-II, and activates ATG5-dependent and LC3B-dependent autophagy of Tau.

### Treatment of cells with myricetin stabilizes the interaction between Tau and ATG5

**Figure 6:**
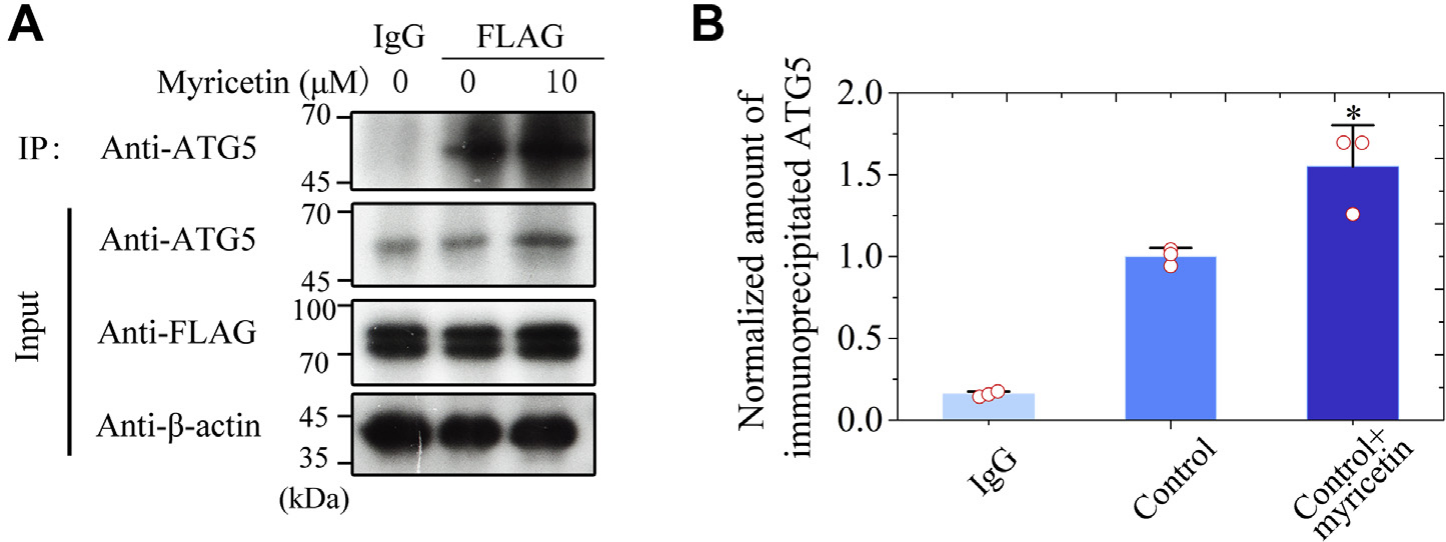
**Treatment of cells with myricetin stabilizes the interaction between Tau and ATG5.***A*, SH-SY5Y cells stably overexpressing Tau were incubated with 10 μM Congo red for 2 days and incubated with 0 μM myricetin or 10 μM myricetin for 2 days. Anti-FLAG binding beads were used for co-IP experiments and then detected by Western blot with anti-ATG5, anti-FLAG, and anti-β-actin antibodies, respectively. *B*, the normalized amount of immunoprecipitated ATG5 in SH-SY5Y cells stably overexpressing Tau (*open red circles* shown in scatter plots) was determined as a ratio of the density of IP:ATG5 bands over the density of β-actin band in cell lysates and expressed as the mean ± S.D. (with error bars) of values obtained in three independent experiments. Control + myricetin, *p* = 0.021. SH-SY5Y cells overexpressing Tau treated without myricetin were used as a control. Statistical analyses were performed using the Student's *t* test. Values of *p* < 0.05 indicate statistically significant differences. The following notation is used throughout: ∗*p* < 0.05; ∗∗*p* < 0.01; and ∗∗∗*p* < 0.001 relative to control.

We have shown that myricetin acts as both an mTOR inhibitor and an ATG5-dependent autophagic activator. We next used co-IP to check whether ATG5 directly interacts with Tau protein to form a complex. The anti-FLAG antibody was used to IP the complex, followed by immunoblotting for ATG5. Notably, our co-IP experiments visualized by anti-ATG5 antibody show that endogenous ATG5 directly interacts with Tau protein in SH-SY5Y cells (Figs. 6*A* and S6, *A–C*). More importantly, we show that treatment of cells with myricetin stabilizes the interaction between Tau and ATG5 (Fig. 6, *A* and *B*; 1.55 ± 0.25 for ATG5−Tau complex + myricetin *versus* 1.00 ± 0.05 for ATG5−Tau complex alone, *p* = 0.021). Our data once again demonstrated that myricetin activated ATG5-dependent Tau autophagy.

### Myricetin activates LC3B-dependent and ATG5-dependent Tau autophagy to promote clearance of phosphorylated Tau

**Figure 7:**
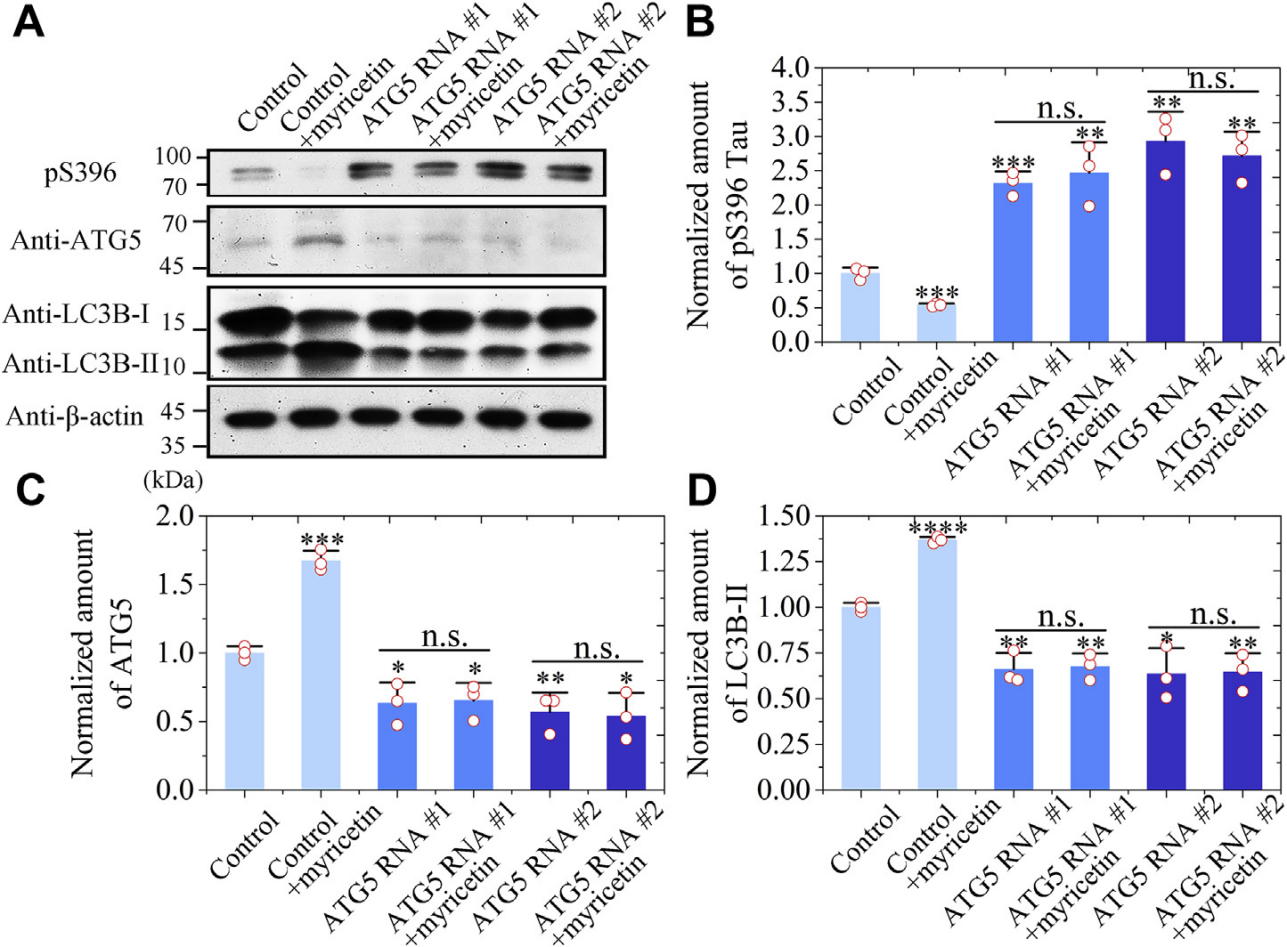
**Myricetin activates LC3B-dependent and ATG5-dependent Tau autophagy to promote clearance of phosphorylated Tau.***A*, SH-SY5Y cells stably overexpressing full-length human Tau were incubated with 10 μM Congo red for 2 days and then incubated with 0 μM myricetin or 10 μM myricetin for 2 days. SH-SY5Y cells stably overexpressing Tau were transfected with ATG5 RNAi #1 and ATG5 RNAi #2, and then the cells were incubated with 0 μM myricetin or 10 μM myricetin for 2 days. The cell lysates were probed with the anti-pS396 antibody, anti-ATG5 antibody, anti-LC3B antibody, and anti-β-actin antibody, respectively. *B*−*D*, the normalized amount of pS396 Tau (*B*), ATG5 (*C*), and LC3B-II (*D*) in SH-SY5Y cells stably overexpressing Tau (*open red circles* shown in scatter plots) was determined as a ratio of the density of pS396 Tau, ATG5, and LC3B-II bands over the density of β-actin band in cell lysates, respectively, and expressed as the mean ± S.D. (with error bars) of values obtained in three independent experiments. *B*, the normalized amount of pS396 Tau in the cell lysates treated with 10 μM myricetin was significantly lower than that in the control cell lysates treated without myricetin (0.539 ± 0.023 for pS396 Tau + myricetin *versus* 1.000 ± 0.087 for pS396 Tau alone). Knockdown of *ATG5* using RNAi #1 and RNAi #2 significantly enhanced the levels of the phosphorylated Tau in the cell lysates (2.32 ± 0.17 for pS396 Tau + RNAi #1 and 2.93 ± 0.43 for pS396 Tau + RNAi #2 *versus* 1.00 ± 0.09 for pS396 Tau + control). Myricetin did not significantly ameliorate pathological phosphorylation of Tau in *ATG5* knockdown SH-SY5Y cells (2.47 ± 0.45 for pS396 Tau + ATG5 RNAi #1 + myricetin *versus* 2.32 ± 0.17 for pS396 Tau + ATG5 RNAi #1; and 2.71 ± 0.35 for pS396 Tau + ATG5 RNAi #2 + myricetin *versus* 2.93 ± 0.43 for pS396 Tau + ATG5 RNAi #2). *C* and *D*, the normalized amounts of ATG5 and LC3B-II in the cell lysates treated with 10 μM myricetin were significantly higher than those in the control cell lysates (1.671 ± 0.075 for ATG5 + myricetin *versus* 1.000 ± 0.050 for ATG5 alone; and 1.368 ± 0.018 for LC3B-II + myricetin *versus* 1.000 ± 0.024 for LC3B-II alone). Knockdown of *ATG5* using RNAi #1 and RNAi #2 significantly reduced the levels of ATG5 and LC3B-II in the cell lysates (0.63 ± 0.15 for ATG5 + RNAi #1 and 0.57 ± 0.14 for ATG5 + RNAi #2 *versus* 1.00 ± 0.05 for ATG5 + control; and 0.66 ± 0.09 for LC3B-II + RNAi #1 and 0.63 ± 0.14 for LC3B-II + RNAi #2 *versus* 1.00 ± 0.02 for LC3B-II + control). Myricetin did not significantly activate ATG5-dependent and LC3B-dependent Tau autophagy when *ATG5* gene was knocked down by RNAi (0.65 ± 0.13 for ATG5 RNAi #1 + myricetin *versus* 0.63 ± 0.15 for ATG5 RNAi #1 alone; 0.54 ± 0.17 for ATG5 RNAi #2 + myricetin *versus* 0.57 ± 0.14 for ATG5 RNAi #2 alone; 0.68 ± 0.07 for LC3B-II RNAi #1 + myricetin *versus* 0.66 ± 0.09 for LC3B-II RNAi #1 alone; and 0.65 ± 0.10 for LC3B-II RNAi #2 + myricetin *versus* 0.63 ± 0.14 for LC3B-II RNAi #2 alone). SH-SY5Y cells overexpressing Tau treated without myricetin were used as a control for (*B*−*D*), and those treated RNAi were used as a control for (*C* and *D*). Statistical analyses were performed using the Student's *t* test. Values of *p* < 0.05 indicate statistically significant differences. The following notation is used throughout: ∗*p* < 0.05; ∗∗*p* < 0.01; ∗∗∗*p* < 0.001; and ∗∗∗∗*p* < 0.0001 relative to control. n.s., no significance.

Finally, we used RNAi to knock down *ATG5*, an important gene in the autophagic machinery, in SH-SY5Y cells stably expressing Tau. The cells were cultured with 10 μM Congo red for 2 days, then transiently expressed a control sequence, ATG5 RNAi #1 or ATG5 RNAi #2, and incubated without or with 10 μM myricetin for 2 days. To gain a quantitative understanding of how myricetin activates Tau autophagy and clears phosphorylated Tau in cells, we detected two autophagy-related proteins ATG5 and LC3B from the above cells using anti-ATG5 antibody and anti-LC3B antibody, respectively, and probed the phosphorylated Tau using anti-pS396 antibody (Figs. 7*A* and S7, *A–C*). The normalized amount of pS396 Tau in the cell lysates treated with 10 μM myricetin was significantly lower than that in the control cell lysates treated without myricetin (*p* = 0.00089) (Fig. 7*B*). Knockdown of *ATG5* using RNAi #1 and RNAi #2, however, significantly enhanced the levels of the phosphorylated Tau in the cell lysates (*p* = 0.00030 and 0.0016) (Fig. 7*B*). More importantly, myricetin did not significantly ameliorate pathological phosphorylation of Tau in *ATG5* knockdown SH-SY5Y cells (*p* = 0.61 and 0.91) (Fig. 7*B*). Thus, treatment of cells with the compound helps clear phosphorylated Tau mainly *via* ATG5-dependent autophagy pathway. The normalized amounts of ATG5 and LC3B-II in the cell lysates treated with 10 μM myricetin were significantly higher than those in the control cell lysates (*p* = 0.00021 and 0.000029) (Fig. 7, *C* and *D*). Knockdown of *ATG5* using RNAi #1 and RNAi #2, however, significantly reduced the levels of ATG5 and LC3B-II in the cell lysates (Fig. 5, *C* and *D*). More importantly, myricetin did not significantly activate ATG5-dependent and LC3B-dependent Tau autophagy when *ATG5* gene was knocked down by RNAi (Fig. 7, *C* and *D*). Our immunoblotting data demonstrated that *ATG5* knockdown in SH-SY5Y cells blocked the inhibitory effect of myricetin on pathological phosphorylation of Tau, suggesting that myricetin regulates degradation of the phosphorylated Tau mainly *via* ATG5-dependent autophagy pathway.

### Treatment of cells with myricetin blocks mitochondrial damage resulting from Tau aggregation

**Figure 8:**
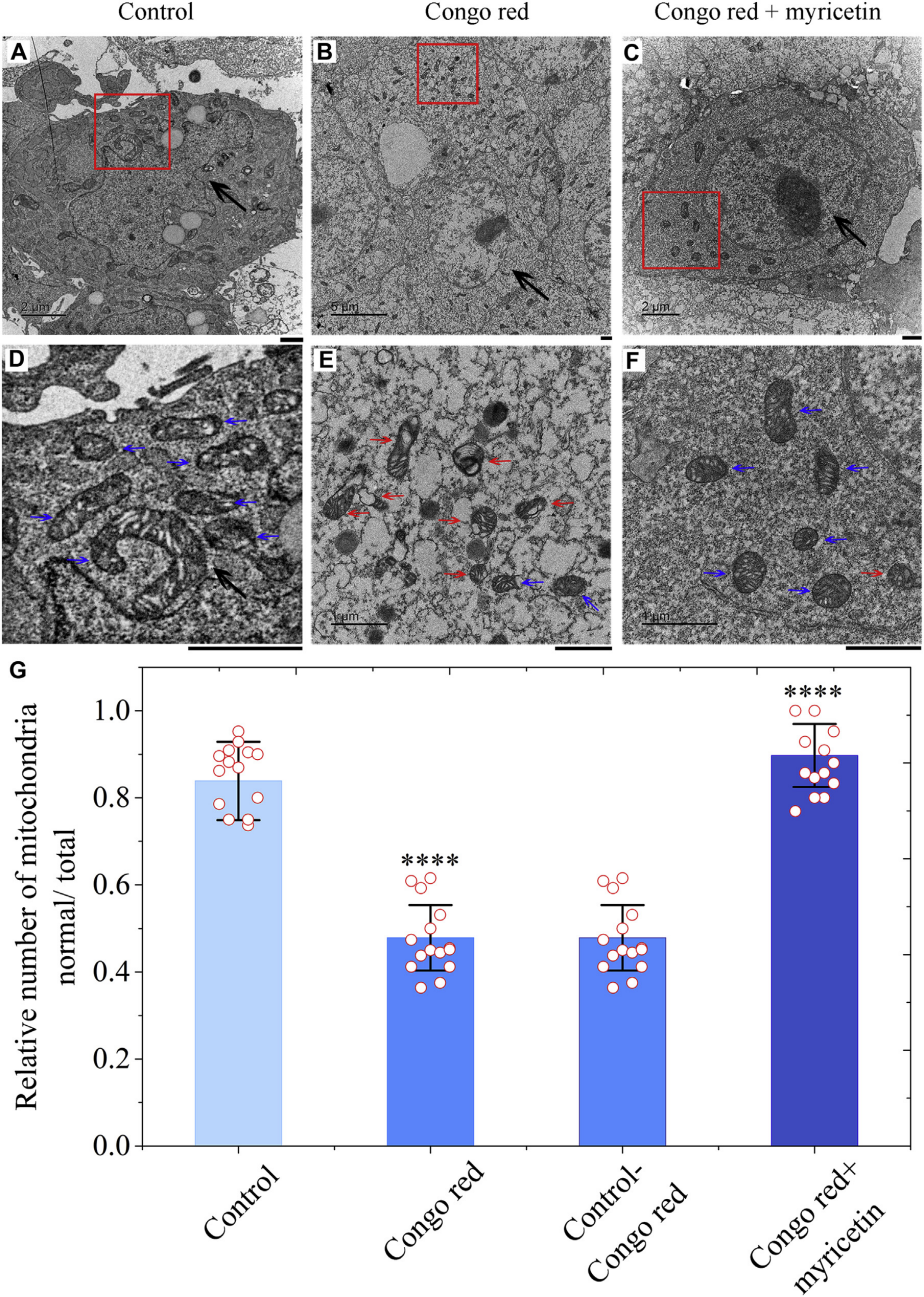
**Treatment of cells with myricetin blocks mitochondrial damage resulting from Tau aggregation.***A*−*F*, SH-SY5Y cell stably overexpressing full-length human Tau were cultured without Congo red (*A* and *D*) or with 10 μM Congo red (*B* and *E*) for 2 days and then incubated with 0 μM myricetin (*A*, *B*, *D*, and *E*) or 10 μM myricetin (*C* and *F*) for 2 days. The enlarged regions (*D*) and (*F*) show 16-fold enlarged images from (*A*) and (*C*), respectively, the enlarged region (*E*) shows 25-fold enlarged image from (*B*), and display the detailed structures of mitochondria in SH-SY5Y cells. Nuclei are highlighted using *black arrows* (*A*−*C*). The morphology of normal mitochondria in SH-SY5Y cells incubated with PBS buffer (*D*) or in SH-SY5Y cells overexpressing Tau incubated with 10 μM Congo red and 10 μM myricetin (*F*), which are highlighted by *blue arrows*, was tubular or round. 10 μM Congo red treatment caused severe mitochondrial impairment in SH-SY5Y cells expressing Tau (*E*). Most of the mitochondria in the cells (∼50%) became swollen and vacuolized, which is highlighted by *red arrows*. Samples were negatively stained using 2% uranyl acetate and lead citrate. The scale bars represent 1 μm. *G*, quantification of TEM images performed on biological replicates shows that myricetin significantly decreases mitochondrial damage resulting from Tau aggregation. The relative number of mitochondria (normal/total) (*open red circles* shown in scatter plots) is expressed as mean ± S.D. (with error bars) of values obtained in three biological replicates. About 30 cells were counted in each group. Tau + Congo red, *p* = 0.000015. SH-SY5Y cells stably overexpressing full-length human Tau treated without Congo red were used as a control. Tau + Congo red + myricetin, *p* = 0.00000098. SH-SY5Y cells stably overexpressing full-length human Tau treated with 10 μM Congo red for 4 days were used as a control. A significantly lower number of normal mitochondria was observed in Congo red-treated cells than did in control cells treated by PBS (0.478 ± 0.011 for Tau + Congo red *versus* 0.859 ± 0.022 for Tau alone). In contrast, a significantly higher number of normal mitochondria was observed in Congo red- and myricetin-treated cells than did in control cells treated by Congo red alone (0.897 ± 0.009 for Tau + Congo red + myricetin *versus* 0.478 ± 0.011 for Tau + Congo red). Statistical analyses were performed using the Student's *t* test. Values of *p* < 0.05 indicate statistically significant differences. The following notation is used throughout: ∗*p* < 0.05; ∗∗*p* < 0.01; ∗∗∗*p* < 0.001; and ∗∗∗∗*p* < 0.0001 relative to control.

Autophagy plays an essential role in protecting cells from mitochondrial damage and cytotoxicity to avoid the development of neurodegenerative diseases (30, 31, 32). We have demonstrated that treatment of cells with myricetin stabilizes the interaction between Tau and ATG5 to promote clearance of phosphorylated Tau to indirectly limit its aggregation. We wonder whether myricetin could block Tau aggregation-induced mitochondrial damage *via* activation of autophagy in tauopathy cells. SH-SY5Y cells stably expressing Tau were cultured with 0 or 10 μM Congo red for 2 days, then incubated with 0 or 10 μM myricetin for 2 days, and investigated by transmission electron microscopy (TEM) (Fig. 8, *A–F*). The morphology of normal mitochondria in cells incubated with PBS buffer, highlighted by blue arrows, was tubular or round (Fig. 8, *A* and *D*). Treatment of Congo red alone caused serious mitochondrial damage in cells stably expressing Tau, half of the mitochondria in the cells became swollen and vacuolized, and cristae rupturing and disappearance were observed, which is highlighted by red arrows (Fig. 8, *B* and *E*). A significantly lower number of normal mitochondria was observed in Congo red-treated cells than did in control cells treated by PBS (*p* = 0.000015) (Fig. 8*G*). In contrast, treatment of Congo red and myricetin together did not cause serious mitochondrial impairment in cells stably expressing Tau and most of the mitochondria were normal in appearance (highlighted by blue arrows) (Fig. 8, *C* and *F*). A significantly higher number of normal mitochondria was observed in Congo red- and myricetin-treated cells than did in control cells treated by Congo red alone (*p* = 0.00000098) (Fig. 8*G*). Therefore, treatment of cells with myricetin blocks mitochondrial damage caused by Tau aggregation and induced by Congo red, possibly *via* activation of autophagy in tauopathy cells.

### Treatment of cells with myricetin suppresses Tau toxicity

**Figure 9:**
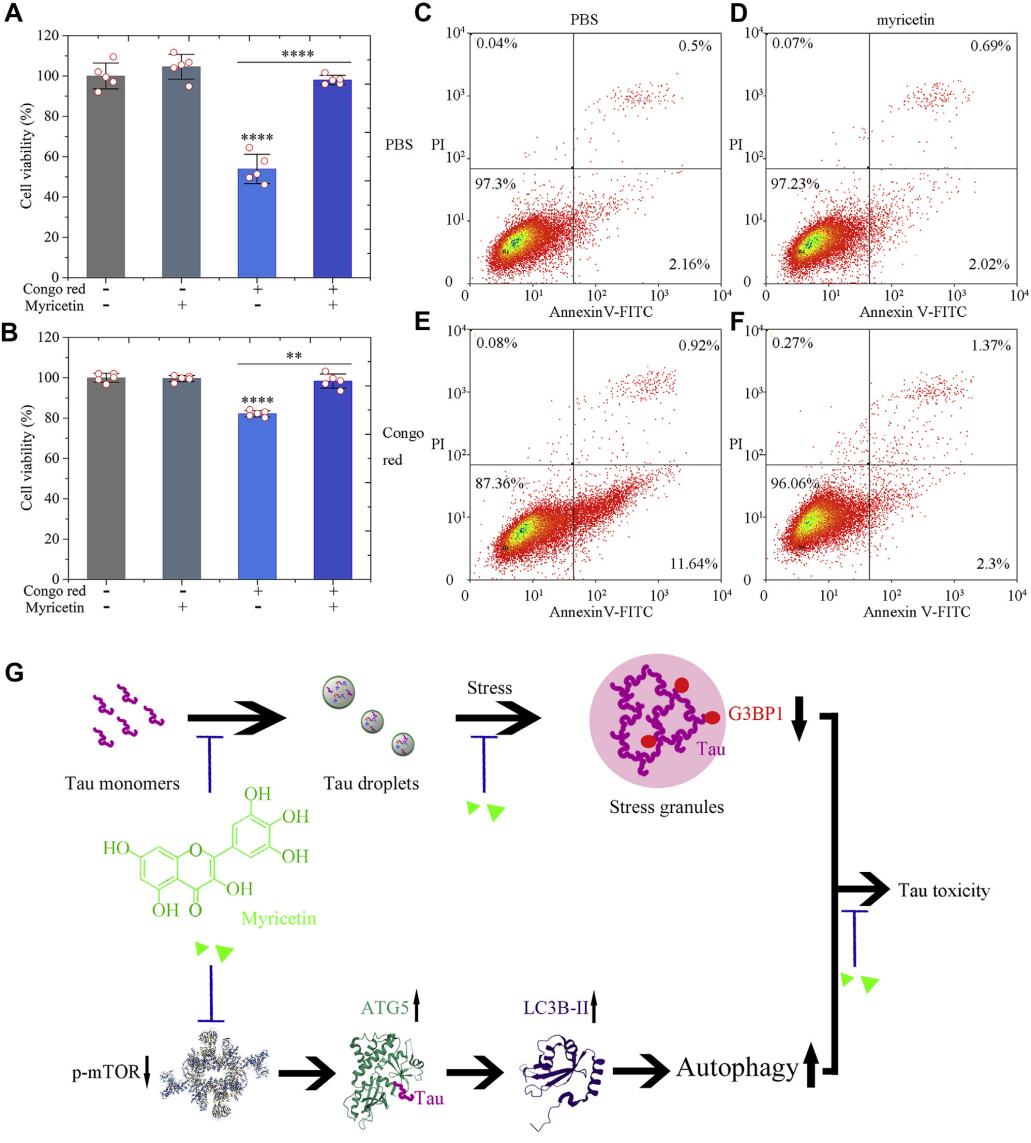
**Myricetin slows down Tau LLPS and suppresses Tau toxicity *via* activating ATG5-dependent Tau autophagy.***A*−*F*, myricetin significantly decreases Tau toxicity in cells. SH-SY5Y cell stably overexpressing Tau were cultured without Congo red or with 10 μM Congo red for 2 days, then incubated with 0 μM or 10 μM myricetin for 2 days and detected by MTT (*A*) and CCK8 (*B*) assays. The cell viability (%) (*open red circles* shown in scatter plots) is expressed as the mean ± S.D. (with error bars) of values obtained in five independent experiments, respectively. Tau + Congo red, *p* = 0.0000058 (*A*) or 0.00000049 (*B*). SH-SY5Y cells stably overexpressing full-length human Tau treated without Congo red were used as a control. Tau + Congo red + myricetin, *p* = 0.0000015 (*A*) or 0.0020 (*B*). SH-SY5Y cells stably overexpressing full-length human Tau treated with 10 μM Congo red for 4 days were used as a control. *A* or *B*, treatment of Congo red alone significantly increased Tau toxicity in Congo red cultured SH-SY5Y cells stably overexpressing Tau (Cell viability: 53.9% ± 7.3% or 82.2% ± 1.5% for Tau + Congo red *versus* 100% ± 6.4% or 100% ± 2.2% for Tau alone). In contrast, treatment of Congo red and myricetin together did not significantly increase Tau toxicity in cells stably overexpressing Tau (Cell viability: 98.0% ± 2.3% or 98.3% ± 3.6% for Tau + Congo red + myricetin *versus* 100% ± 6.4% or 100% ± 2.2% for Tau alone). Myricetin significantly decreased Tau toxicity in SH-SY5Y cells induced by Congo red (Cell viability: 98.0% ± 2.3% or 98.3% ± 3.6% for Tau + Congo red + myricetin *versus* 53.9% ± 7.3% or 82.2% ± 1.5% for Tau + Congo red). Statistical analyses were performed using the Student's *t* test. Values of *p* < 0.05 indicate statistically significant differences. The following notation is used throughout: ∗*p* < 0.05; ∗∗*p* < 0.01; ∗∗∗*p* < 0.001; and ∗∗∗∗*p* < 0.0001 relative to control. SH-SY5Y cell stably overexpressing Tau were cultured without Congo red (*C*, *D*) or with 10 μM Congo red (*E*, *F*) for 2 days and then incubated with 0 μM myricetin (*C*, *E*) or 10 μM myricetin (*D*, *F*) for 2 days. Treatment of 10 μM Congo red (*E*) for 2 days did induce early apoptosis of living Tau stable cells, but no apoptosis of living Tau stable cells was observed when treated with 10 μM Congo red for 2 days and then incubated with 10 μM myricetin (*F*) for 2 days. The percentage of apoptotic cells was determined by flow cytometry. The four quadrants distinguished by annexin V-FITC/PI staining represent viable cells (*lower left quadrant*), early apoptotic cells (*lower right quadrant*), late apoptotic cells (*upper left quadrant*), and operation-damaged cells (*upper left quadrant*). *G*, a hypothetical model shows how myricetin (*green triangles*), a natural flavonoid, slows down Tau LLPS (Tau droplets, *green balls*) and suppresses Tau toxicity *via* activating ATG5-dependent Tau autophagy. More importantly, treatment of cells with myricetin stabilizes the interaction between Tau (*magenta ropes*) and ATG5 (PDB 4TQO) (shown in ribbon representation in *dark green*) to promote clearance of phosphorylated Tau to limit its aggregation. This natural flavonoid inhibits mTOR (PDB 5EF5) (*ribbon structure*) pathway, activates LC3B (PDB 5GMV) (shown in ribbon representation in *purple*)-dependent and ATG5-dependent Tau autophagy, and almost completely suppresses Tau toxicity.

SH-SY5Y cells stably expressing Tau were cultured with 0 or 10 μM Congo red for 2 days, then incubated with 0 or 10 μM myricetin for 2 days, and further investigated by MTT reduction assay, CCK8 reduction assay, and flow cytometry with annexin V-FITC and propidium iodide (PI) staining (17, 45, 47) (Fig. 9, *A–F*). Treatment of Congo red alone significantly increased Tau toxicity in Congo red cultured SH-SY5Y cells stably expressing Tau (from 0 to 46.1% or from 0 to 17.8%; *p* = 0.0000058 or 0.00000049) (Fig. 9, *A* or *B*). In contrast, treatment of Congo red and myricetin together did not significantly increase Tau toxicity in cells stably expressing Tau (from 0 to 2.0% or from 0 to 1.7%; *p* = 0.52 or 0.39) (Fig. 9, *A* or *B*). Myricetin significantly decreased Tau toxicity in SH-SY5Y cells induced by Congo red (*p* = 0.0000015 or 0.0020) (Fig. 9, *A* or *B*). Treatment of Congo red alone also significantly increased the percentage of early apoptotic cells in Congo red cultured SH-SY5Y cells stably expressing Tau (from 2.16% to 11.64%) (Fig. 9, *C* and *E*). However, myricetin significantly decreased the percentage of early apoptotic cells in Congo red cultured SH-SY5Y cells stably expressing Tau (from 11.64% to 2.3%) (Fig. 9, *E* and *F*). Furthermore, our control experiments demonstrated that 10 μM myricetin alone had no obvious toxicity to SH-SY5Y cells stably expressing Tau (Fig. 9, *A*, *B*, and *D*). Together, these results demonstrate that treatment of cells with myricetin almost completely suppresses Tau toxicity induced by Congo red, possibly *via* activation of autophagy in tauopathy cells.

## Discussion

Recently, various *in vitro* assays have confirmed that certain polyphenols have the ability to inhibit amyloid fibril formation of some key proteins linked to neurodegenerative diseases such as tauopathies (22, 23, 24, 25, 26, 48), but the mechanisms behind the phenomenon are still not clear. We now show that myricetin slows LLPS of full-length Tau protein and significantly inhibits the subsequent Tau aggregation. One striking observation is that myricetin modulates material properties of full-length Tau protein from liquid droplets to solid aggregates during the conversion of Tau LLPS into aggregation. Accumulating evidences point to a crucial role of phase transitions in neurodegenerative diseases associated with protein aggregation and misregulation of membrane-less organelles (49, 50, 51, 52, 53, 54). Liquid-phase condensation of some key proteins linked to neurodegenerative diseases is modulated by disease-associated modifications, molecular chaperones, and other factors (12, 13, 14, 15, 16, 17, 18, 19, 20, 21, 55). Therefore, we studied the role of myricetin in regulating the formation of stress granules containing Tau in neuronal cells. Notably, we show that myricetin reduces the colocalization of Tau and stress granules and prevents the accumulation of Tau in stress granules. Furthermore, myricetin inhibits pathological phosphorylation and aggregation of Tau protein in neuronal cells. Intriguingly, the interaction of Tau protein with TIA1 has been found to regulate stress granule formation as well as misfolding and aggregation of Tau (53, 54).

In the present study, we described the influences of the natural antioxidant flavonoid compound myricetin on the concentration dependence of droplet formation of full-length Tau protein *in vitro* as well as stress granule formation by Tau, treatment studies of Tau expressing cells that model mitochondrial damage, and Congo-red-induced Tau phosphorylation and toxicity. We demonstrated that myricetin clears phosphorylated Tau aggregates and reduces Tau toxicity *via* activating ATG5-dependent Tau autophagy in cells. We also observed that myricetin slows down LLPS of full-length Tau *in vitro* and stress granule formation of Tau in cells. The phase separation and turbidity measurements were performed under reducing conditions to ensure that the protein did not form disulfide-linked oligomers. Myricetin slows down LLPS and enhances fluorescence recovery of full-length Tau protein under reducing conditions, possibly *via* interaction with the protein to lessen intermolecular interactions in Tau condensates.

One of the challenging problems is to understand how myricetin promotes degradation of intracellular Tau aggregates. Autophagy is thought to play an important role in the catabolism of pathological Tau, which has aroused interest in developing autophagy-based therapeutics in tauopathies (56, 57). Autophagy stimulation could be performed either using small-molecule enhancers of autophagy or *via* gene therapy approaches (39, 58, 59, 60). We observed decreased levels of phosphorylated mTOR, an indicator of repressive mTOR signaling, and increased levels of LC3B-II and ATG5-ATG12 complex upon myricetin treatment, indicating that treatment of cells with the compound triggers mTOR inhibition and autophagic activation. More importantly, we show that Tau is as an interacting partner of ATG5, and treatment of cells with myricetin stabilizes intermolecular electrostatic interactions between the positively charged middle/C-terminal regions of Tau and the negatively charged ATG5. It is well known that ATG5 is required for autophagosome formation and is essential for autophagy (30, 36). Intriguingly, we observed that the autophagy level was decreased in ATG5 RNAi cells, which blocked the inhibitory effect of pathologically phosphorylated Tau upon myricetin treatment. These cumulative results point to a critical role of mTOR pathway and ATG5-dependent Tau autophagy in the cellular mechanisms through which myricetin could trigger degradation of Tau aggregates.

In summary, we report a natural flavonoid (antioxidant compound) myricetin that slows LLPS of full-length Tau protein *in vitro*. In particular, our results show a mild effect of myricetin on Tau LLPS, so one is left to wonder if the observed data add much to the cellular story that is provided next. How does the fact that LLPS of full-length Tau protein is mildly shifted by myricetin impact on the cellular assays that follow? To address this question, we suggest that the retardation of Tau LLPS under reducing conditions might lead to inhibition of stress granule formation, activation of autophagy function, and less Tau fibril formation in cells. Our data generally supports the claims and are sufficient to show that Tau LLPS and aggregation are slowed down by the compound. Our results describe a model to underpin molecular hypotheses of how myricetin can slow down Tau LLPS (Tau liquid phase condensation) and ameliorate Tau toxicity *via* activating ATG5-dependent Tau autophagy (Fig. 9*G*). Importantly, we show that treatment of cells with myricetin stabilizes the interaction between Tau and ATG5 to promote clearance of phosphorylated Tau aggregates to limit its aggregation and association with stress granules and that the effect of the compound is indirect. This natural flavonoid inhibits mTOR pathway, activates LC3B-dependent and ATG5-dependent Tau autophagy, and almost completely suppresses Tau toxicity in neuronal cells (Fig. 9*G*). These results are of interest to the protein folding community as it attempts to bridge the gap between Tau LLPS and aggregation in neuronal cells. Furthermore, we could search for therapeutic targets of neurodegenerative diseases through the combination of amyloid fibril structures (61, 62, 63) and natural flavonoids. Very recently, phase-separated droplets have been found to be specific autophagy substrates in cells, which are modulated by the interplay between liquid droplets and membrane sheets of autophagosomes (64). Future research on catabolism stress granules with aberrant Tau through myricetin-modulated autophagy pathway to test whether Tau mediates the interplay between stress granules and autophagosomes will highlight an innovative and facile strategy against Alzheimer's disease and the related tauopathies.

## Experimental procedures

### Materials

Congo red (fresh molecular weight of 696.67), DAPI, the mouse anti-FLAG antibody (F1804), mouse anti-β-actin antibody (A1978), rabbit anti-ATG5 antibody (SAB5700062), rabbit anti-LC3B antibody (SAB1306269), rabbit anti-mTOR antibody (SAB4501038), and rabbit anti-p-mTOR antibody (SAB5700327) were purchased from Sigma-Aldrich, rabbit anti-pS396 polyclonal antibody (ab32057) was purchased from Abcam, and rabbit/mouse Anti-G3BP1 polyclonal antibody was purchased from Proteintech (13057-2-AP/66486-1-Ig). All Alexa-conjugated fluorescent secondary antibodies (rabbit A0208; mouse A0216) and cell lysis buffer for western and IP were purchased from Beyotime. SP Sepharose Fast Flow was purchased from GE Company. All other chemicals used in this study were of analytical grade and were produced in China. A plasmid-encoding human Tau40 was a kind gift from Dr Michel Goedert (University of Cambridge), and a plasmid-encoding G3BP1 was a kind gift from Dr Liu Yong (Wuhan University).

### Tau protein expression and purification

A prokaryotic expression vector pRK172-encoding human Tau40 was a kind gift from Dr Michel Goedert (University of Cambridge). The construction of prokaryotic plasmids expressing full-length human Tau and Tau protein purification were carried as described (10, 11, 17, 45). Purified Tau protein was analyzed by SDS-PAGE with one band. The concentration of human Tau was determined according to its absorbance at 214 nm with a standard calibration curve drawn by BSA.

### Liquid-droplet formation

The freshly purified full-length human Tau protein was incubated with TAMRA (red fluorescence, excitation at 546 nm) at a Tau: TAMRA molar ratio of 1:3 for 1 h. These labeled proteins were filtered and freeze-dried. In total, 5.0, 10.0, 15.0, and 20.0 μM Tau protein labeled by TAMRA were incubated with 10 mM HEPES buffer (pH 7.4) containing 10% (w/v) PEG 4000 and 2 mM β-mercaptoethanol or incubated with the same buffer further containing 10.0 μM myricetin on 25 °C for 5 min to induce LLPS. Liquid droplets of Tau were observed by a Leica TCS SP8 laser scanning confocal microscope with excitation at 546 nm. All phase separation experiments were performed at least three times and were quite reproducible.

### Turbidity assays

In total, 0, 2.5, 5.0, 7.5, 10.0, 12.5, 15.0, 17.5, and 20 μM full-length human Tau labeled by TAMRA were incubated with 10 mM HEPES buffer (pH 7.4) containing 10% (w/v) PEG 4000 and 2 mM β-mercaptoethanol or incubated with the same buffer further containing 10.0 μM myricetin at 25 °C for 5 min to induce LLPS. The turbidity of Tau condensates was measured at 400 nm and 25 °C using a Cytation 3 Cell Imaging Multi-Mode Reader (BioTek), and turbidity changes for Tau LLPS in the absence of myricetin were used as controls. All turbidity assays were repeated at least three times. Statistical analyses were performed using the Student's *t* test. Values of *p* < 0.05 indicate statistically significant differences. The following notation is used throughout: ∗*p* < 0.05; ∗∗*p* < 0.01; and ∗∗∗*p* < 0.001 relative to controls.

### Fluorescence recovery after photobleaching (FRAP)

In total, 20 μM full-length human Tau labeled by TAMRA was incubated with 10 mM HEPES buffer (pH 7.4) containing 10% (w/v) PEG 4000 and 2 mM β-mercaptoethanol or incubated with the same buffer further containing 10 μM myricetin at 25 °C to induce LLPS for 30 min. Liquid droplets of full-length Tau were observed by a Zeiss LSM 880 laser scanning microscope (Carl Zeiss) with excitation at 546 nm. For each droplet, a square was bleached at 80% transmission for 2 s, and postbleaching time-lapse images were collected (35 frames, 6 s per frame). Images were analyzed using Zen (LSM 880 confocal microscope manufacturer's software).

### Cell culture and transfection

SH-SY5Y neuroblastoma cells were cultured in minimum essential media and in Dulbecco's modified Eagle's medium (Gibco, Invitrogen), supplemented with 10% (v/v) fetal bovine serum (Gibco), 100 U/ml streptomycin, and 100 U/ml penicillin in 5% CO_2_ at 37 °C. SH-SY5Y cell line stably expressing FLAG-tagged full-length human Tau or Tau-EGFP was constructed with a lentiviral vector construction system (pHAGE-puro). The target DNA fragments were inserted into the lentiviral vector, and the plasmids containing target DNA, pVSVG, and p976 were packaged in HEK-293T cells at a ratio of 2:1:1 by Lipofectamine 2000 (Invitrogen). The ratio of liposome to DNA was 2:1. After 48 h of transfection, the viruses were harvested and filtered, and then SH-SY5Y cells were infected with the packaged lentivirus twice for 12 h each with a 12-h interval. In order to establish the stable cell lines, puromycin was used to screen overexpressed cells. The expression of each protein was detected by Western blot.

### Western blotting

SH-SY5Y cells stably expressing full-length human Tau were cultured with 10 μM Congo red in 6-well plates for 2 days and then cultured with 0 or 10 μM myricetin for 2 days. The cells were harvested and ruptured on ice for 15 min with RIPA lysis buffer containing 50 mM Tris (pH 7.4), 150 mM NaCl, 1% NP-40, 0.5% sodium deoxycholate, 0.1% SDS, EDTA, and leupeptin (Beyotime). The cell lysates were boiled in SDS-PAGE loading buffer for 10 min, then subjected to 12.5% SDS-PAGE for Figures 4*A*, 5*I*, 6*A*, and 7*A* or 8% SDS-PAGE for Figure 5*K*, and transferred to polyvinylidene difluoride membranes (Millipore). The membranes were blocked with 5% fat-free milk in 25 mM Tris-buffered saline buffer containing 0.047% Tween 20 (TBST) and then incubated with an appropriate dilution ratio of the following primary antibodies at 4 °C overnight, *i.e.*, rabbit anti-pS396 antibody for Figure 4*A*, rabbit anti-LC3B and ra`bbit anti-ATG5 antibodies for Figure 5*I*, rabbit anti-mTOR and rabbit anti-p-mTOR antibodies for Figure 5*K*, and mouse anti-β-actin antibody for Figures 4, *A* and *C*, and 5, *I* and *K*, followed by incubation with 1/10,000 homologous horseradish peroxidase (HRP)-conjugated secondary antibody for 1 h at room temperature. The membranes were then incubated with WesternBright ECL HRP substrate (Advansta Inc) and developed on films. The normalized amounts of pS396 Tau, ATG5, LC3B-II, or mTOR/p-mTOR in SH-SY5Y cells stably expressing full-length Tau were determined as a ratio of the density of pS396 Tau, ATG5, LC3B-II, or mTOR/p-mTOR bands over the density of β-actin band in the cell lysates and are expressed as mean ± SD (with error bars) of values obtained in three independent experiments. SH-SY5Y cells overexpressing Tau treated without myricetin were used as a control. For calculating the amounts of pS396 Tau, ATG5, LC3B-II, or mTOR/p-mTOR, the ImageJ software (NIH) was used to assess the densitometry of the corresponding protein bands. Statistical analyses were performed using the Student's *t* test. Values of *p* < 0.05 indicate statistically significant differences. The following notation is used throughout: ∗*p* < 0.05; ∗∗*p* < 0.01; ∗∗∗*p* < 0.001; and ∗∗∗∗*p* < 0.0001 relative to control.

### Sarkosyl-insoluble western blotting

Sarkosyl-insoluble Western blotting was used to investigate intracellular Tau aggregates. Cell lysates from the above SH-SY5Y stable cells were centrifuged at 17,000 *g* for 30 min at 4 °C to remove the cell debris. Half of the supernatant was incubated with 1% sarkosyl for 30 min at 25 °C. The mixture was then ultracentrifuged at 150,000 *g* for 30 min, and the pellets were washed twice with 1 × PBS (pH 7.4). The sarkosyl-insoluble pellets were boiled in the SDS-PAGE loading buffer for 10 min. The other half of the supernatant, which served as the total protein sample, was also boiled in the SDS-PAGE loading buffer for 10 min. The samples were separated by 12.5% SDS-PAGE and then Western blotted as described in detail in the aforementioned Tau phosphorylation experiments. The sarkosyl-insoluble pellets from those cells were probed using the anti-FLAG antibody, and the corresponding cell lysates were probed using the anti-FLAG antibody and anti-β-actin antibody. The amount of loaded protein was normalized using a BCA Protein Quantification kit (Beyotime). For calculating the amounts of sarkosyl-insoluble Tau, the ImageJ software (NIH) was used to assess the densitometry of Tau bands. The normalized amount of insoluble Tau aggregates in SH-SY5Y cells stably expressing full-length Tau was determined as a ratio of the density of Tau aggregate bands over the density of β-actin band in the cell lysates. SH-SY5Y cells overexpressing Tau treated without myricetin were used as a control. Statistical analyses were performed using the Student's *t* test. Values of *p* < 0.05 indicate statistically significant differences. The following notation is used throughout: ∗*p* < 0.05; ∗∗*p* < 0.01; and ∗∗∗*p* < 0.001 relative to control.

### Laser scanning confocal analysis

SH-SY5Y cells stably expressing FLAG-tagged full-length human Tau were cultured with 10 μM Congo red in 12-well plates for 2 days and then cultured with 0 or 10 μM myricetin for 2 days. Cells were fixed, permeabilized, immunostained with primary antibodies anti-LC3B and anti-FLAG, and then immunostained with second antibody IgG conjugated to Alexa Fluor 488 (green) and IgG conjugated to Alexa Fluor 555 (red), respectively. Images were captured using a Leica TCS SP8 laser scanning confocal microscope.

### Stress granule formation and 3D rendering

SH-SY5Y cells transiently expressing full-length human Tau labeled by EGFP (or stably overexpressing FLAG-tagged full-length human Tau) with endogenous G3BP1 were incubated with 10 μM Congo red in 12-well plates for 2 days, cultured without myricetin or with 10 μM myricetin for 2 days, and then incubated 500 μM sodium arsenite for 45 min at 37 °C. Cells were fixed with 4% paraformaldehyde for 30 min, followed by lysed in 0.25% Triton X-100 and blocked with 5% BSA at 37 °C, immunostained with primary antibody rabbit anti-G3BP1 (or primary antibody mouse anti-FLAG), and then immunostained with second antibody IgG conjugated to Alexa Fluor 555 (or second antibody IgG conjugated to Alexa Fluor 488). Images of Tau-EGFP (green) and G3BP1 immunostained by Alexa Fluor 555 (red) were captured using a Zeiss LSM 880 Laser Scanning Microscope (Carl Zeiss). A stack (20–30 slices) of optical sections were acquired at 0.10 μm (Z steps) increments with XY pixel dimensions of 66 × 66 μm and obtained to reveal the 3D image of the above SH-SY5Y cells. All images were processed for line intensity profile analysis in the programs Zen (LSM 880 confocal microscope manufacturer's software). Images of FLAG-tagged full-length Tau (green) and G3BP1 immunostained by Alexa Fluor 555 (red) were captured using a Leica TCS SP8 laser scanning confocal microscope.

### Coimmunoprecipitation

SH-SY5Y cells stably expressing full-length human Tau were cultured with 10 μM Congo red in 6-well plates for 2 days and then cultured with 0 or 10 μM myricetin for 2 days. Cells were harvested and ruptured on ice for 15 min. Three hundred and fifty microliters of the cell lysates from the above cells was mixed with 3.5 μl of a cocktail protease inhibitors, immunoprecipitated with 1 μl of 1.0 mg/ml mouse anti-FLAG overnight at 4 °C, and then incubated with 20 μl of Protein G Agarose beads for 4 h at 4 °C. The beads were washed five times with PBS buffer, boiled in SDS-PAGE loading buffer for 10 min, and then probed with Western blot using rabbit anti-ATG5 (the upper lane). Nonspecific IgG was served as a negative control in immunoprecipitation. Another 50 μl of cell lysates was boiled in SDS-PAGE loading buffer, probed with Western blot using anti-ATG5 antibody, anti-FLAG antibody, and mouse anti-β-actin, respectively, and served as the input controls (the middle lane and the lower lane), which represented the total ATG5 content, the total Tau content, and the total protein content in cell lysates, respectively.

### ATG5 RNAi

pLKO.1 DNA constructs expressing a specific shRNA were from the MISSION TRC-Hs 1.0 library. Constructs including control: TTCTCCGAACGTGTCACGTTT; ATG 5 RNAi #1: AATATCTCATCCTGATATAGC; and ATG 5 RNAi #2: AAATGAGCTTCAATTGCATCC were obtained as bacterial clones. Transient knockdown experiments with pLKO.1-ATG5 RNAi were conducted by using Lipofectamine 2000 (Invitrogen) according to the manufacturer's reverse transfection protocol. SH-SY5Y cells stably expressing full-length human Tau were cultured with 10 μM Congo red in 6-well plates for 2 days. In each well of a 6-well plate, 2 ng pLKO.1-ATG 5 RNAi control #1 or # 2 and 10 μl Lipofectamine 2000 were diluted in 200 μl Opti-MEM, and the transfection solution was incubated in 5% CO_2_ at 37 °C for 4–6 h and then cultured in minimum essential media with 0 or 10 μM myricetin for 2 days. Knockdown events were verified by Western blotting with rabbit anti-pS396, rabbit anti-ATG5, rabbit anti-LC3B, and mouse β-actin antibodies.

### Ultrathin TEM

SH-SY5Y cells stably expressing full-length human Tau were cultured with 10 μM Congo red in 6-well plates for 2 days and then cultured with 0 or 10 μM myricetin for 2 days, and cells cultured with PBS alone as a negative control. SH-SY5Y cells stably expressing full-length human Tau incubated with PBS were used as a negative control. After prefixation with 3% paraformaldehyde and 1.5% glutaraldehyde in 1 × PBS (pH 7.4), the cells were harvested and postfixed in 1% osmium tetroxide for 1 h using an ice bath; the samples were then dehydrated in graded acetone and embedded in 812 resins. Ultrathin sections of the cells were prepared using a Leica Ultracut S Microtome and negatively stained using 2% uranyl acetate and lead citrate. The doubly stained ultrathin sections of cells were examined using a JEM-1400 Plus transmission electron microscope (JEOL) operating at 80 kV.

### Cell proliferation and cytotoxicity assay

SH-SY5Y cells stably expressing full-length human Tau were cultured with 10 μM Congo red in 96-well plates for 2 days and then cultured with 10 μM myricetin for 2 days. SH-SY5Y cells stably expressing full-length human Tau treated without Congo red were used as a control. Or SH-SY5Y cells stably expressing full-length human Tau treated with 10 μM Congo red for 4 days without myricetin were used as a control. Cells were incubated in new medium containing 0.5 mg/ml MTT for a further 4 h, and then the medium was replaced with 150 μl of pure dimethyl sulfoxide, and the absorbance of the dark blue formazan was measured with a microplate reader at 492 nm. Or cells were incubated in new medium containing 10% Cell Counting Kit-8 (CCK8) for 2 h, and the absorbance of the orange formazan was measured with a microplate reader at 450 nm.

### Annexin V-FITC apoptosis detection assay

SH-SY5Y cells stably expressing full-length human Tau were cultured without Congo red or with 10 μM Congo red for 2 days and then incubated with 0 μM myricetin or 10 μM myricetin for 2 days. Apoptotic cells were detected by flow cytometry after staining with an annexin V-FITC apoptosis detection kit (Beyotime). In brief, SH-SY5Y cells were harvested after digestion with 2.5 mg/ml trypsin (Promega); the cells were washed twice with 1 × PBS at 4 °C and resuspended in 195 μl of binding buffer. The samples were then incubated with 5 μl of annexin V-FITC and 10 μl of PI for 15 min at 4 °C in the dark. Annexin V binding was analyzed using an EPICS XL-MCL flow cytometer (Beckman Coulter), and the percentage of apoptotic cells was calculated from the total number of cells (∼2 × 10^4^ cells) using EXPO32MultiComp software. All apoptotic blot experiments were repeated at least three times.

### Statistical analysis

The data shown for each experiment were based on at least three technical replicates, as indicated in individual figure legends. Data are presented as mean ± SD, and *p*-values were determined using the Student's *t* test. All experiments were further confirmed by biological repeats.

## Data availability

All data generated or analyzed during this study are included in this published article or available from the corresponding author upon request.

## Supporting information

This article contains supporting information.

## Conflict of interest

The authors declare that they have no conflicts of interest with the contents of this article.

## References

[1] Guo T., Noble W., Hanger D.P. (2017). Roles of Tau protein in health and disease. Acta Neuropathol..

[2] Rauch J.N., Olson S.H., Gestwicki J.E. (2017). Interactions between microtubule-associated protein Tau (MAPT) and small molecules. Cold Spring Harb. Perspect. Med..

[3] Kellogg E.H., Hejab N.M.A., Poepsel S., Downing K.H., DiMaio F., Nogales E. (2018). Near-atomic model of microtubule-Tau interactions. Science.

[4] Baas P.W., Qiang L. (2019). Tau: it's not what you think. Trends Cell Biol..

[5] Mukrasch M.D., Bibow S., Korukottu J., Jeganathan S., Biernat J., Griesinger C., Mandelkow E., Zweckstetter M. (2009). Structural polymorphism of 441-residue Tau at single residue resolution. PLoS Biol..

[6] Mandelkow E.M., Mandelkow E. (2012). Biochemistry and cell biology of Tau protein in neurofibrillary degeneration. Cold Spring Harb. Perspect. Med..

[7] Alzheimer's Association (2020). 2020 Alzheimer's disease facts and figures. Alzheimers Dement..

[8] Wang Y., Mandelkow E. (2016). Tau in physiology and pathology. Nat. Rev. Neurosci..

[9] Grundke-Iqbal I., Iqbal K., Tung Y.C., Quinlan M., Wisniewski H.M., Binder L.I. (1986). Abnormal phosphorylation of the microtubule-associated protein Tau (Tau) in Alzheimer cytoskeletal pathology. Proc. Natl. Acad. Sci. U. S. A..

[10] Zhu H.L., Fernández C., Fan J.B., Shewmaker F., Chen J., Minton A.P., Liang Y. (2010). Quantitative characterization of heparin binding to Tau protein: Implication for inducer-mediated Tau filament formation. J. Biol. Chem..

[11] Liu X.L., Hu J.Y., Hu M.Y., Zhang Y., Hong Z.Y., Cheng X.Q., Chen J., Pang D.W., Liang Y. (2015). Sequence-dependent abnormal aggregation of human Tau fragment in an inducible cell model. Biochim. Biophys. Acta.

[12] Wegmann S., Eftekharzadeh B., Tepper K., Zoltowska K.M., Bennett R.E., Dujardin S., Laskowski P.R., MacKenzie D., Kamath T., Commins C., Vanderburg C., Roe A.D., Fan Z.Y., Molliex A.M., Hernandez-Vega A. (2018). Tau protein liquid-liquid phase separation can initiate Tau aggregation. EMBO J..

[13] Mathieu C., Pappu R.V., Taylor J.P. (2020). Beyond aggregation: Pathological phase transitions in neurodegenerative disease. Science.

[14] Zhang X., Lin Y., Eschmann N.A., Zhou H., Rauch J.N., Hernandez I., Guzman E., Kosik K.S., Han S. (2017). RNA stores Tau reversibly in complex coacervates. PLoS Biol..

[15] Ukmar-Godec T., Hutten S., Grieshop M.P., Rezaei-Ghaleh N., Cima-Omori M., Biernat J., Mandelkow E., Söding J., Dormann D., Zweckstetter M. (2019). Lysine/RNA-interactions drive and regulate biomolecular condensation. Nat. Commun..

[16] Babinchak W.M., Dumm B.K., Venus S., Boyko S., Putnam A.A., Jankowsky E., Surewicz W.K. (2020). Small molecules as potent biphasic modulators of protein liquid-liquid phase separation. Nat. Commun..

[17] Wang K., Liu J.Q., Zhong T., Liu X.L., Zeng Y., Qiao X.H., Xie T., Chen Y.Z., Gao Y.Y., Tang B., Li J., Zhou J., Pang D.W., Chen J., Chen C. (2020). Phase separation and cytotoxicity of Tau are modulated by protein disulfide isomerase and S-nitrosylation of this molecular chaperone. J. Mol. Biol..

[18] Kanaan N.M., Hamel C., Grabinski T., Combs B. (2020). Liquid-liquid phase separation induces pathogenic Tau conformations *in vitro*. Nat. Commun..

[19] Boyko S., Surewicz K., Surewicz W.K. (2020). Regulatory mechanisms of Tau protein fibrillation under the conditions of liquid-liquid phase separation. Proc. Natl. Acad. Sci. U. S. A..

[20] Singh V., Xu L., Boyko S., Surewicz K., Surewicz W.K. (2020). Zinc promotes liquid-liquid phase separation of Tau protein. J. Biol. Chem..

[21] Ambadipudi S., Biernat J., Riedel D., Mandelkow E., Zweckstetter M. (2017). Liquid-liquid phase separation of the microtubule-binding repeats of the Alzheimer-related protein Tau. Nat. Commun..

[22] Landau M., Sawaya M.R., Faull K.F., Laganowsky A., Jiang L., Sievers S.A., Liu J., Barrio J.R., Eisenberg D. (2011). Towards a pharmacophore for amyloid. PLoS Biol..

[23] Bulic B., Pickhardt M., Mandelkow E. (2013). Progress and developments in Tau aggregation inhibitors for Alzheimer disease. J. Med. Chem..

[24] Bu X.L., Rao P.P.N., Wang Y.J. (2016). Anti-amyloid aggregation activity of natural compounds: Implications for Alzheimer's drug discovery. Mol. Neurobiol..

[25] Umeda T., Ono K., Sakai A., Yamashita M., Mizuguchi M., Klein W.L., Yamada M., Mori H., Tomiyama T. (2016). Rifampicin is a candidate preventive medicine against amyloid-β and Tau oligomers. Brain.

[26] Zhu M., Rajamani S., Kaylor J., Han S., Zhou F., Fink A.L. (2004). The flavonoid baicalein inhibits fibrillation of α-synuclein and disaggregates existing fibrils. J. Biol. Chem..

[27] Semwal D.K., Semwal R.B., Combrinck S., Viljoen A. (2016). Myricetin: A dietary molecule with diverse biological activities. Nutrients.

[28] Simunkova M., Alwasel S.H., Alhazza I.M., Jomova K., Kollar V., Rusko M., Valko M. (2019). Management of oxidative stress and other pathologies in Alzheimer's disease. Arch. Toxicol..

[29] Hasima N., Ozpolat B. (2014). Regulation of autophagy by polyphenolic compounds as a potential therapeutic strategy for cancer. Cell Death Dis..

[30] Mizushima N., Komatsu M. (2011). Autophagy: Renovation of cells and tissues. Cell.

[31] Galluzzi L., Baehrecke E.H., Ballabio A., Boya P., Bravo-San Pedro J.M., Cecconi F., Choi A.M., Chu C.T., Codogno P., Colombo M.I., Cuervo A.M., Debnath J., Deretic V., Dikic I., Eskelinen E.L. (2017). Molecular definitions of autophagy and related processes. EMBO J..

[32] Galves M., Rathi R., Prag G., Ashkenazi A. (2019). Ubiquitin signaling and degradation of aggregate-prone proteins. Trends Biochem. Sci..

[33] Scrivo A., Bourdenx M., Pampliega O., Cuervo A.M. (2018). Selective autophagy as a potential therapeutic target for neurodegenerative disorders. Lancet Neurol..

[34] Djajadikerta A., Keshri S., Pavel M., Prestil R., Ryan L., Rubinsztein D.C. (2020). Autophagy induction as a therapeutic strategy for neurodegenerative diseases. J. Mol. Biol..

[35] Kerr J.S., Adriaanse B.A., Greig N.H., Mattson M.P., Cader M.Z., Bohr V.A., Fang E.F. (2017). Mitophagy and Alzheimer's disease: Cellular and molecular mechanisms. Trends Neurosci..

[36] Nakatogawa H., Suzuki K., Kamada Y., Ohsumi Y. (2009). Dynamics and diversity in autophagy mechanisms: Lessons from yeast. Nat. Rev. Mol. Cell Biol..

[37] Zhao Y.G., Zhang H. (2019). Core autophagy genes and human diseases. Curr. Opin. Cell Biol..

[38] Mizushima N., Yoshimori T., Levine B. (2010). Methods in mammalian autophagy research. Cell.

[39] Noda N.N., Inagaki F. (2015). Mechanisms of autophagy. Annu. Rev. Biophys..

[40] Fujioka Y., Alam J.M., Noshiro D., Mouri K., Ando T., Okada Y., May A.I., Knorr R.L., Suzuki K., Ohsumi Y., Noda N.N. (2020). Phase separation organizes the site of autophagosome formation. Nature.

[41] Webb J.L., Ravikumar B., Atkins J., Skepper J.N., Rubinsztein D.C. (2003). α-Synuclein is degraded by both autophagy and the proteasome. J. Biol. Chem..

[42] Berger Z., Ravikumar B., Menzies F.M., Oroz L.G., Underwood B.R., Pangalos M.N., Schmitt I., Wullner U., Evert B.O., O'Kane C.J., Rubinsztein D.C. (2006). Rapamycin alleviates toxicity of different aggregate-prone proteins. Hum. Mol. Genet..

[43] Alberti S., Gladfelter A., Mittag T. (2019). Considerations and challenges in studying liquid-liquid phase separation and biomolecular condensates. Cell.

[44] Yang P., Mathieu C., Kolaitis R.M., Zhang P., Messing J., Yurtsever U., Yang Z., Wu J., Li Y., Pan Q., Yu J., Martin E.W., Mittag T., Kim H.J., Taylor J.P. (2020). G3BP1 is a tunable switch that triggers phase separation to assemble stress granules. Cell.

[45] Hu J.Y., Zhang D.L., Liu X.L., Li X.S., Cheng X.Q., Chen J., Du H.N., Liang Y. (2017). Pathological concentration of zinc dramatically accelerates abnormal aggregation of full-length human Tau and thereby significantly increases Tau toxicity in neuronal cells. Biochim. Biophys. Acta.

[46] Lira-De Leon K.I., Garcia-Gutierrez P., Serratos I.N., Palomera-Cardenas M., Figueroa-Corona Mdel P., Campos-Pena V., Meraz-Rios M.A. (2013). Molecular mechanism of Tau aggregation induced by anionic and cationic dyes. J. Alzheimers Dis..

[47] Xu W.C., Liang J.Z., Li C., He Z.X., Yuan H.Y., Huang B.Y., Liu X.L., Tang B., Pang D.W., Du H.N., Yang Y., Chen J., Wang L., Zhang M., Liang Y. (2018). Pathological hydrogen peroxide triggers the fibrillization of wild-type SOD1 via sulfenic acid modification of Cys-111. Cell Death Dis..

[48] Lunven L., Bonnet H., Yahiaoui S., Yi W., Da Costa L., Peuchmaur M., Boumendjel A., Chierici S. (2016). Disruption of fibers from the Tau model AcPHF6 by naturally occurring aurones and synthetic analogues. ACS Chem. Neurosci..

[49] Boyko S., Qi X., Chen T.H., Surewicz K., Surewicz W.K. (2019). Liquid-liquid phase separation of Tau protein: The crucial role of electrostatic interactions. J. Biol. Chem..

[50] Guo L., Kim H.J., Wang H., Monaghan J., Freyermuth F., Sung J.C., O'Donovan K., Fare C.M., Diaz Z., Singh N., Zhang Z.C., Coughlin M., Sweeny E.A., DeSantis M.E., Jackrel M.E. (2018). Nuclear-import receptors reverse aberrant phase transitions of RNA-binding proteins with prion-like domains. Cell.

[51] Nedelsky N.B., Taylor J.P. (2019). Bridging biophysics and neurology: Aberrant phase transitions in neurodegenerative disease. Nat. Rev. Neurol..

[52] Protter D.S.W., Parker R. (2016). Principles and properties of stress granules. Trends Cell Biol..

[53] Vanderweyde T., Apicco D.J., Youmans-Kidder K., Ash P.E.A., Cook C., Lummertz da Rocha E., Jansen-West K., Frame A.A., Citro A., Leszyk J.D., Ivanov P., Abisambra J.F., Steffen M., Li H., Petrucelli L. (2016). Interaction of Tau with the RNA-binding protein TIA1 regulates Tau pathophysiology and toxicity. Cell Rep..

[54] Apicco D.J., Ash P.E.A., Maziuk B., LeBlang C., Medalla M., Al Abdullatif A., Ferragud A., Botelho E., Ballance H.I., Dhawan U., Boudeau S., Cruz A.L., Kashy D., Wong A., Goldberg L.R. (2018). Reducing the RNA binding protein TIA1 protects against Tau-mediated neurodegeneration *in vivo*. Nat. Neurosci..

[55] Qamar S., Wang G., Randle S.J., Ruggeri F.S., Varela J.A., Lin J.Q., Phillips E.C., Miyashita A., Williams D., Strohl F., Meadows W., Ferry R., Dardov V.J., Tartaglia G.G., Farrer L.A. (2018). FUS phase separation is modulated by a molecular chaperone and methylation of arginine cation-π interactions. Cell.

[56] Silva J.M., Rodrigues S., Sampaio-Marques B., Gomes P., Neves-Carvalho A., Dioli C., Soares-Cunha C., Mazuik B.F., Takashima A., Ludovico P., Wolozin B., Sousa N., Sotiropoulos I. (2019). Dysregulation of autophagy and stress granule-related proteins in stress-driven Tau pathology. Cell Death Differ..

[57] Shin J.H., Park S.J., Jo D.S., Park N.Y., Kim J.B., Bae J.E., Jo Y.K., Hwang J.J., Lee J.A., Jo D.G., Kim J.C., Jung Y.K., Koh J.Y., Cho D.H. (2019). Down-regulated TMED10 in Alzheimer disease induces autophagy via ATG4B activation. Autophagy.

[58] Fernández Á.F., Sebti S., Wei Y., Zou Z., Shi M., McMillan K.L., He C., Ting T., Liu Y., Chiang W.C., Marciano D.K., Schiattarella G.G., Bhagat G., Moe O.W., Hu M.C. (2018). Disruption of the beclin 1-BCL2 autophagy regulatory complex promotes longevity in mice. Nature.

[59] Zhang X., Li L., Chen S., Yang D., Wang Y., Zhang X., Wang Z., Le W. (2011). Rapamycin treatment augments motor neuron degeneration in SOD1(G93A) mouse model of amyotrophic lateral sclerosis. Autophagy.

[60] Perera N.D., Sheean R.K., Lau C.L., Shin Y.S., Beart P.M., Horne M.K., Turner B.J. (2018). Rilmenidine promotes MTOR-independent autophagy in the mutant SOD1 mouse model of amyotrophic lateral sclerosis without slowing disease progression. Autophagy.

[61] Wang L.Q., Zhao K., Yuan H.Y., Wang Q., Guan Z., Tao J., Li X.N., Sun Y., Yi C.W., Chen J., Li D., Zhang D., Yin P., Liu C., Liang Y. (2020). Cryo-EM structure of an amyloid fibril formed by full-length human prion protein. Nat. Struct. Mol. Biol..

[62] Fitzpatrick A.W.P., Falcon B., He S., Murzin A.G., Murshudov G., Garringer H.J., Crowther R.A., Ghetti B., Goedert M., Scheres S.H.W. (2017). Cryo-EM structures of Tau filaments from Alzheimer's disease. Nature.

[63] Shi Y., Murzin A.G., Falcon B., Epstein A., Machin J., Tempest P., Newell K.L., Vidal R., Garringer H.J., Sahara N., Higuchi M., Ghetti B., Jang M.K., Scheres S.H.W., Goedert M. (2021). Cryo-EM structures of Tau filaments from Alzheimer's disease with PET ligand APN-1607. Acta Neuropathol..

[64] Agudo-Canalejo J., Schultz S.W., Chino H., Migliano S.M., Saito C., Koyama-Honda I., Stenmark H., Brech A., May A.I., Mizushima N., Knorr R.L. (2020). Wetting regulates autophagy of phase-separated compartments and the cytosol. Nature.

