# Supplementary material for: Myricetin slows liquid–liquid phase separation of Tau and activates ATG5-dependent autophagy to suppress Tau toxicity
Source: J Biol Chem. 2021 Sep 22;297(4):101222. doi: 10.1016/j.jbc.2021.101222 (PMC8551527; doi:10.1016/j.jbc.2021.101222)
Supplement: Figures S1–S7 [file mmc1.pdf]

Myricetin slows liquid-liquid phase separation of Tau and activates ATG5-dependent autophagy to suppress Tau toxicity

Bin Dai, Tao Zhong, Zhi-Xian Chen, Wang Chen, Na Zhang, Xiao-Ling Liu, Li-Qiang Wang, Jie Chen, and Yi Liang[1]

From the Hubei Key Laboratory of Cell Homeostasis, College of Life Sciences, Wuhan University, Wuhan 430072, China, and the Wuhan University Shenzhen Research Institute, Shenzhen 518057, China

Running Title: Myricetin suppresses Tau phase separation and toxicity

[1] To whom correspondence should be addressed. Tel.: 86-27-6875-4902; Fax: 86-27-6875-4902; E-mail: liangyi@whu.edu.cn.

Keywords: Tau protein, Protein aggregation, Protein liquid-liquid phase separation, Myricetin, Alzheimer's disease, Tau toxicity

Supplemental Data

Figure S1. Myricetin slows down the formation of stress granules containing Tau in a much larger number of cells. SH-SY5Y cells stably overexpressing FLAG-tagged full-length human Tau with endogenous G3BP1 were incubated with 10 μM Congo red for 2 days, cultured without myricetin (*A*–*D*) or with 10 μM myricetin (*E*–*H*) for 2 days, then incubated 500 μM sodium arsenite for 45 min, fixed, permeabilized, immunostained with mouse anti-FLAG antibody (*green*) and the anti-G3BP1 (*red*) antibody, and observed by confocal microscopy. The confocal microscopy images (*B*, *C*, *F*, and *G*) show that myricetin significantly inhibits the formation of stress granules containing Tau in a much larger number of cells. *White arrows* were used to highlight G3BP1-positive stress granules containing Tau in SH-SY5Y cells (*B*–*D*) and the merge images (*D* and *H*) display that myricetin significantly reduces co-localization of Tau and stress granules (*D*, yellow dots) in SH-SY5Y cells. SH-SY5Y cells with empty vector and endogenous G3BP1 were incubated with 10 μM Congo red for 2 days, cultured without myricetin (*I*–*L*) or with 10 μM myricetin (*M*–*P*) for 2 days, then incubated 500 μM sodium arsenite for 45 min, fixed, permeabilized, immunostained with mouse anti-FLAG antibody and the anti-G3BP1 (*red*) antibody, and observed by confocal microscopy. *Red dots* indicated G3BP1-positive stress granules formed in SH-SY5Y cells under stress conditions (*C*, *G*, *K*, and *O*). The confocal microscopy images (*K* and *O*) and the merge images (*L* and *P*) show that myricetin has no impact on stress granule formation in a much larger number of SH-SY5Y cells with empty vector. Nuclei were visualized by DAPI (*blue*). The scale bars represent 10 μm.

Figure S2. Quantification of images of G3BP1-positive stress granules show that myricetin slows down the formation of stress granules containing Tau in a much larger number of cells. Histograms show the distribution of cell counts and the number of stress granules per cell incubated with 10 μM Congo red and cultured without myricetin (*A* and *C*) or with 10 μM myricetin (*B* and *D*) in SH-SY5Y cells stably overexpressing FLAG-tagged full-length human Tau with endogenous G3BP1 (*A* and *B*) or in SH-SY5Y cells with empty vector and endogenous G3BP1 (*C* and *D*, control). About 70 cells were counted in each group. 1.18 ± 1.01 stress granules per cell for Tau + myricetin versus 2.69 ± 1.40 stress granules per cell for Tau alone; and 2.60 ± 1.43 stress granules per cell for control + myricetin versus 2.71 ± 1.44 stress granules per cell for control. Evaluation and quantification of images of G3BP1-positive stress granules performed on three biological replicates show that myricetin significantly reduces the number of stress granules containing Tau in a much larger number of SH-SY5Y cells stably overexpressing Tau but has no impact on the number of stress granules in a much larger number of SH-SY5Y cells with empty vector. Statistical analyses were performed using the Student's *t* test. Values of $p < 0.05$ indicate statistically significant differences.

Figure S3. Full-length blots and gels of full-length human Tau in cells. These images come from the same blot (*A*, *B*, *C*, *D*, *E* or *F*) and show that myricetin significantly inhibits pathological phosphorylation (*A*−*C*) and aggregation (*D*–*F*) of Tau protein in cells. (*A*–*C*) The cell lysates from the cells in Figure 4*A* were probed by the anti-pS396 antibody and anti-β-actin antibody, respectively. The enlarged regions from *A* (the red boxes) show the detailed bands of human Tau in Figure 4*A*, and *B* and *C* represent two of the biological replicates of *A*. (*D*–*F*) The sarkosyl-insoluble pellets from the cells in Figure 4*C* were probed using anti-FLAG antibody and the corresponding cell lysates were probed using anti-FLAG antibody and anti-β-actin antibody, respectively. The enlarged regions from *D* (the red boxes) show the detailed bands of human Tau in Figure 4*C*, and *E* and *F* represent two of the biological replicates of *D*.

Figure S4. Control experiments that monitor Tau in the absence of Congo red. SH-SY5Y cells stably overexpressing full-length human Tau were incubated without Congo red for 2 days and then cultured with 0 or 10 μM myricetin for 2 days. The sarkosyl-insoluble pellets from the above cells were probed using anti-FLAG antibody and the corresponding cell lysates were probed using anti-FLAG antibody and anti-β-actin antibody, respectively. Full-length blots and gels of FLAG-tagged Tau in cells. In the absence of Congo red, we did not observe Tau aggregates in the detergent-insoluble pellets when the cells were treated with 10 μM myricetin or treated without myricetin. All blots also show the position of the molecular-weight markers.

Figure S5. Full-length blots and gels of mTOR, ATG5 and LC3B in cells. These images come from the same blot (*A* or *B*) and show that myricetin inhibits mTOR pathway (*B*) and activates ATG5-dependent Tau autophagy (*A*). (*A*) The cell lysates from the cells in Figure 5*I* were probed by the anti-ATG5 antibody, anti-LC3B antibody, and anti-β-actin antibody, respectively. The enlarged regions from *A* (the red boxes) show the detailed bands of ATG5 and LC3B in Figure 5*I* with an exposed time of 1 s. (*B*) The cell lysates from the cells in Figure 5*K* were probed by the anti-mTOR antibody, anti-p-mTOR antibody, and anti-β-actin antibody, respectively. The enlarged regions from *B* (the red boxes) show the detailed bands of mTOR and p-mTOR in Figure 5*K* with an exposed time of 60 s.

Figure S6. Full-length blots and gels for co-IP experiments of full-length human Tau with ATG5 in cells. These images come from the same blot (*A*, *B* or *C*) and show that treatment of cells with myricetin stabilizes interaction between Tau and ATG5. (*A*) SH-SY5Y cells stably overexpressing Tau were incubated with 10 μM Congo red for 2 days and incubated with 0 μM myricetin or 10 μM myricetin for 2 days. Anti-FLAG binding beads were used for co-IP experiments and then detected by Western blot with anti-ATG5, anti-FLAG, and anti-β-actin antibodies, respectively. The enlarged regions from *A* (the red boxes) show the detailed bands of immunoprecipitated ATG5 and human Tau in Figure 6*A*, and *B* and *C* represent two

of the biological replicates of *A*.

Figure S7. Full-length blots and gels of phosphorylated Tau, ATG5 and LC3B in cells. These images come from the same blot (*A*, *B* or *C*) and show that myricetin activates LC3B-dependent and ATG5-dependent Tau autophagy to help clear phosphorylated Tau. (*A*) SH-SY5Y cells stably overexpressing full-length human Tau were incubated with 10 μM Congo red for 2 days and then incubated with 0 μM myricetin or 10 μM myricetin for 2 days. The cells in Figure 7*A* were transfected with ATG5 RNAi #1 and ATG5 RNAi #2, and then the cells were incubated with 0 μM myricetin or 10 μM myricetin for 2 days. The cell lysates were probed with the anti-pS396 antibody, anti-ATG5 antibody, anti-LC3B antibody, and anti-β-actin antibody, respectively. The enlarged regions from *A* (the red boxes) show the detailed bands of phosphorylated Tau, ATG5 and LC3B in Figure 7*A* with an exposed time of 20 s, and *B* and *C* represent two of the biological replicates of *A*.

Figure S1

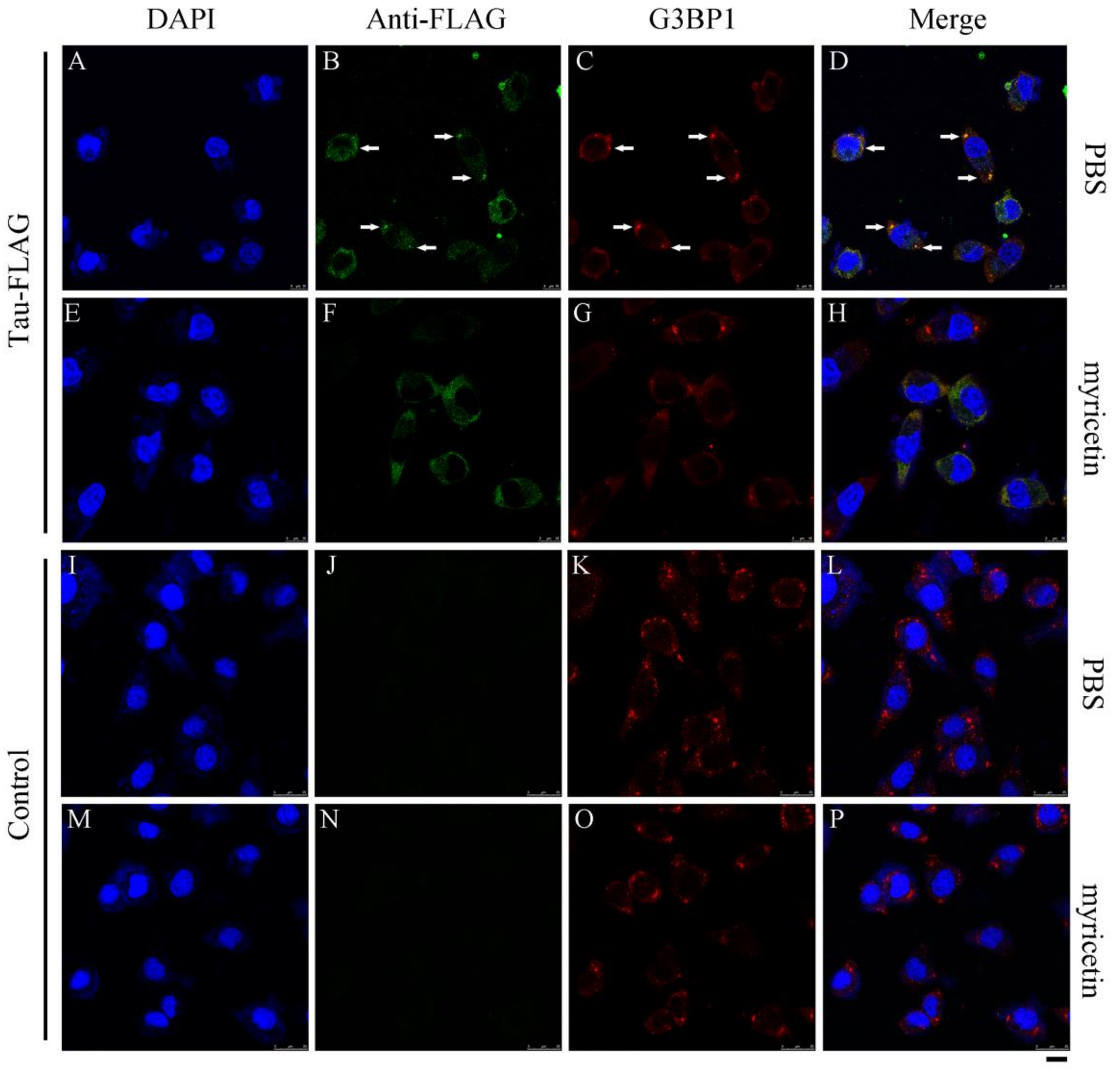

DAPI
Anti-FLAG
G3BP1
Merge
Tau-FLAG
Control
PBS
myricetin
PBS
myricetin
A
B
C
D
E
F
G
H
I
J
K
L
M
N
O
P

Figure S2

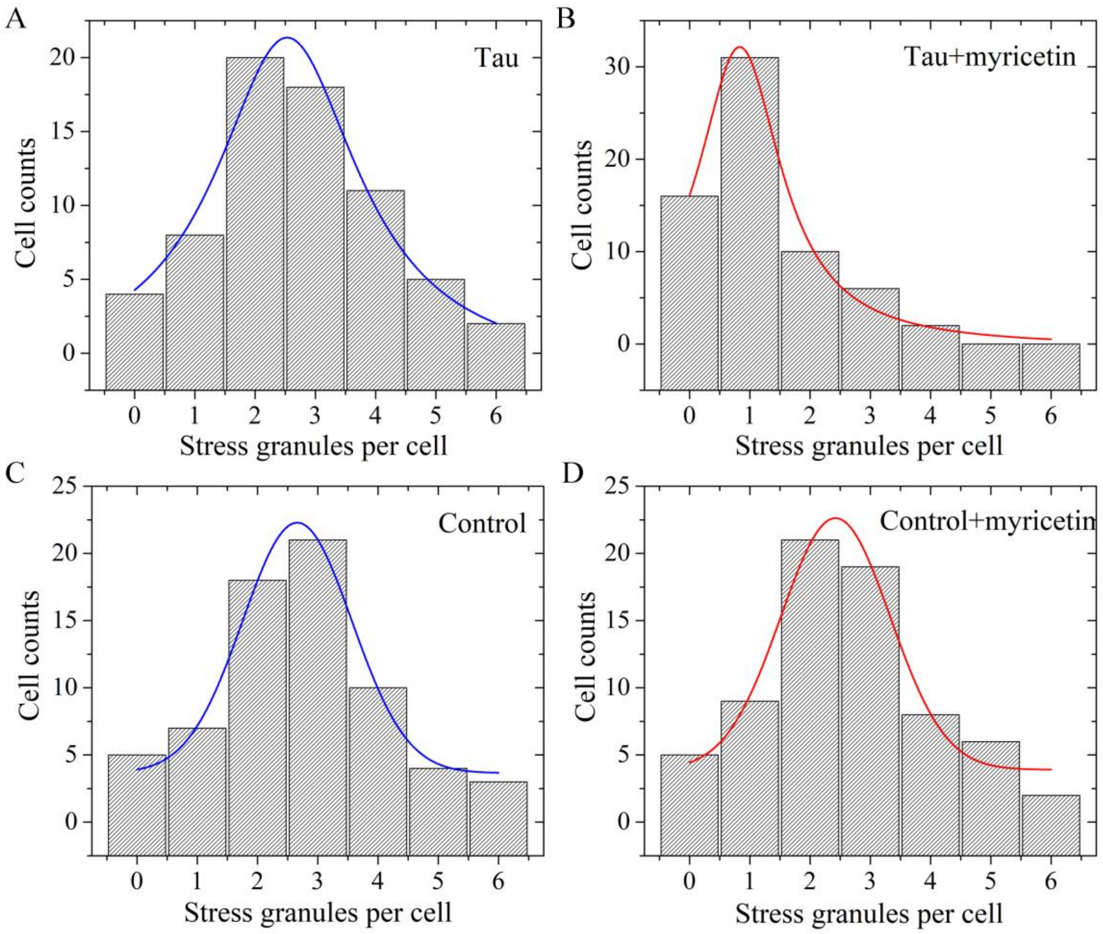
A
Tau
B
Tau+myricetin
C
Control
D
Control+myricetin
Cell counts
Stress granules per cell

Figure S3

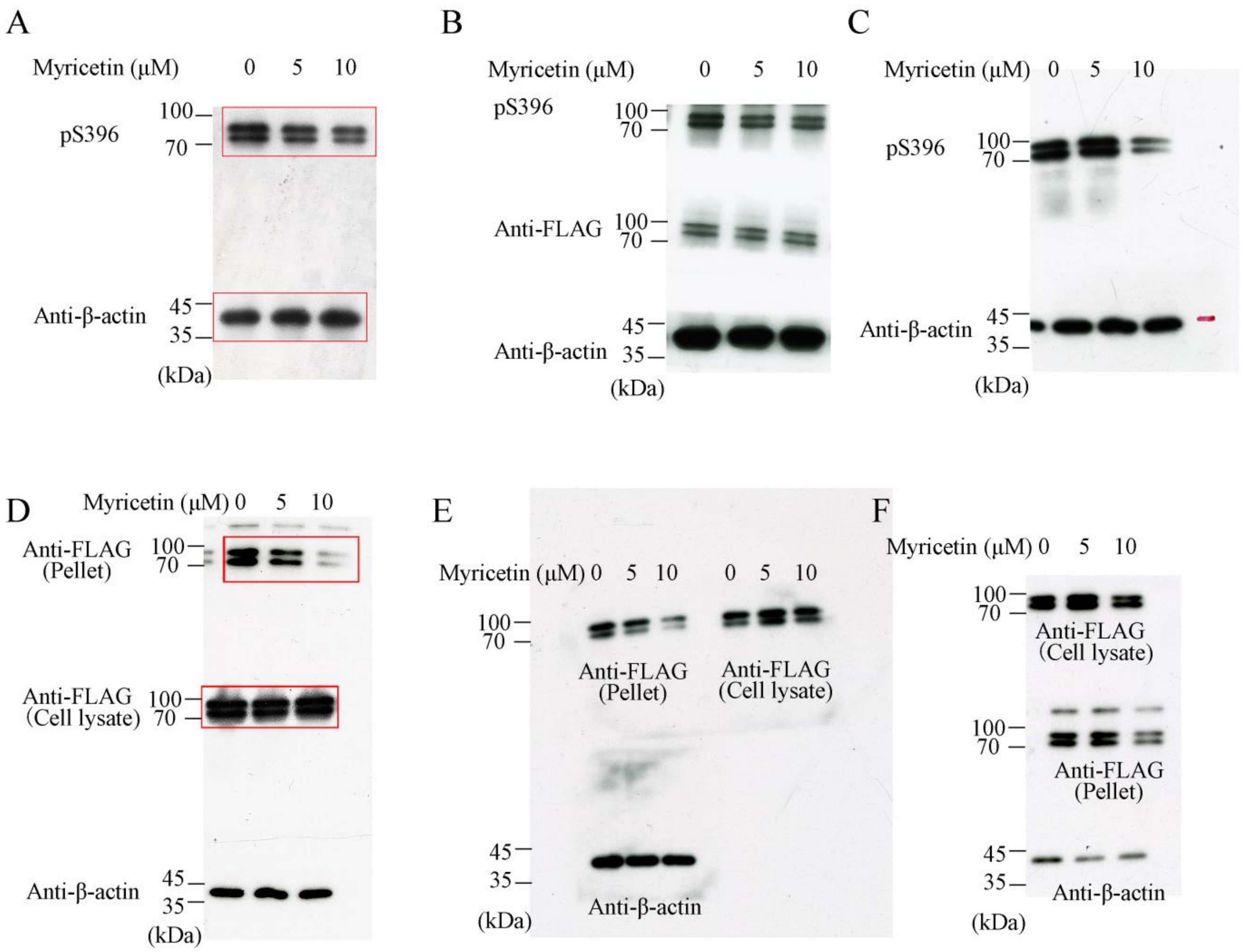
A
Myricetin (μM) 0 5 10
pS396
Anti-β-actin
(kDa)
B
Myricetin (μM) 0 5 10
pS396
Anti-FLAG
Anti-β-actin
(kDa)
C
Myricetin (μM) 0 5 10
pS396
Anti-β-actin
(kDa)
D
Myricetin (μM) 0 5 10
Anti-FLAG (Pellet)
Anti-FLAG (Cell lysate)
Anti-β-actin
(kDa)
E
Myricetin (μM) 0 5 10 0 5 10
Anti-FLAG (Pellet)
Anti-FLAG (Cell lysate)
Anti-β-actin
(kDa)
F
Myricetin (μM) 0 5 10
Anti-FLAG (Cell lysate)
Anti-FLAG (Pellet)
Anti-β-actin
(kDa)

Figure S4

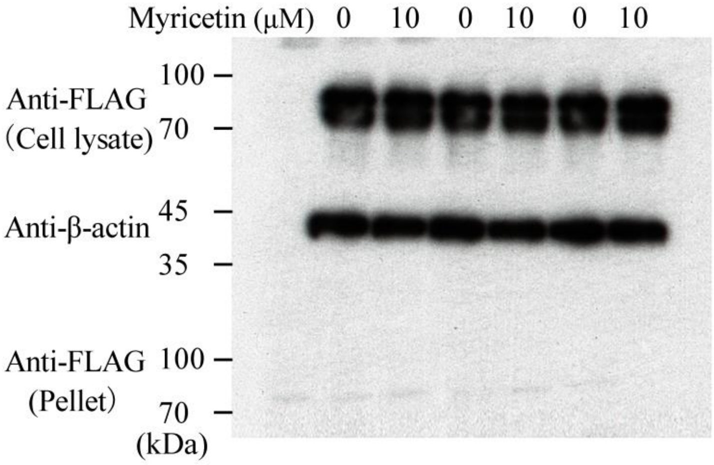
Myricetin (μM) 0 10 0 10 0 10
Anti-FLAG
(Cell lysate)
Anti-β-actin
Anti-FLAG
(Pellet)
100
70
45
35
100
70
(kDa)

Figure S5

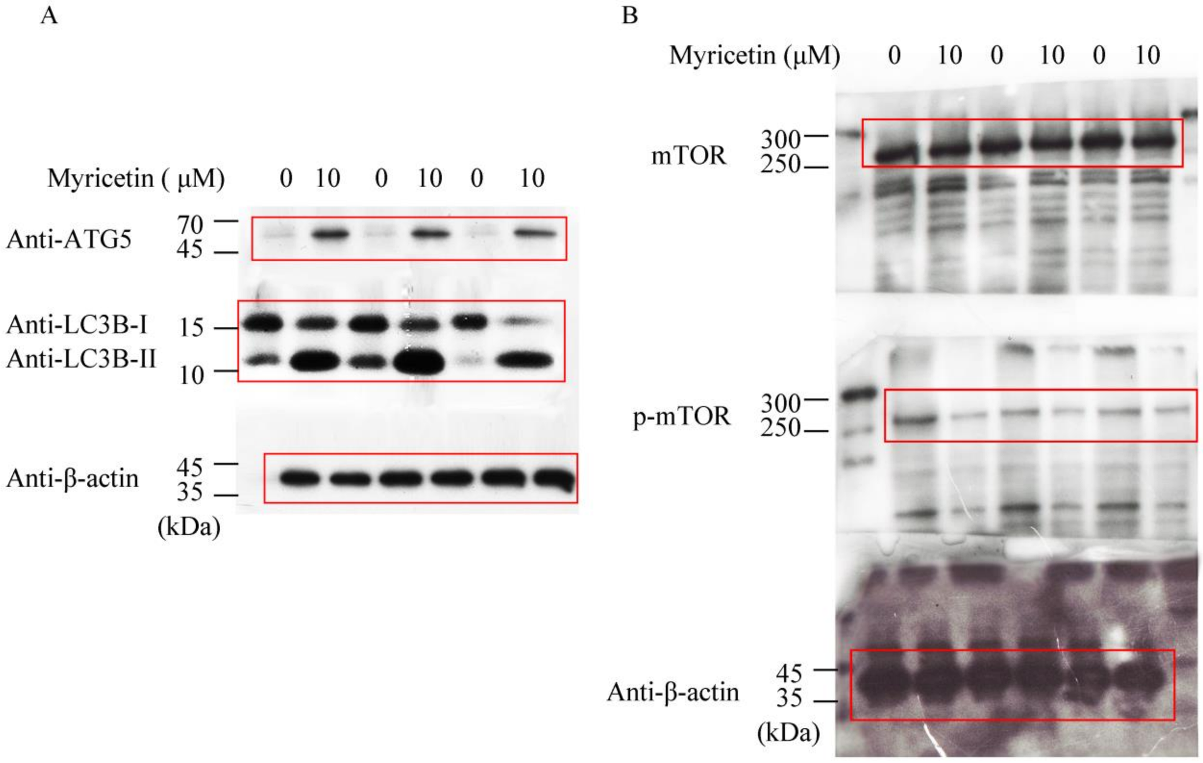

A
Myricetin (μM) 0 10 0 10 0 10
Anti-ATG5
70
45
Anti-LC3B-I
Anti-LC3B-II
15
10
Anti-β-actin
45
35
(kDa)
B
Myricetin (μM) 0 10 0 10 0 10
mTOR
300
250
p-mTOR
300
250
Anti-β-actin
45
35
(kDa)

Figure S6

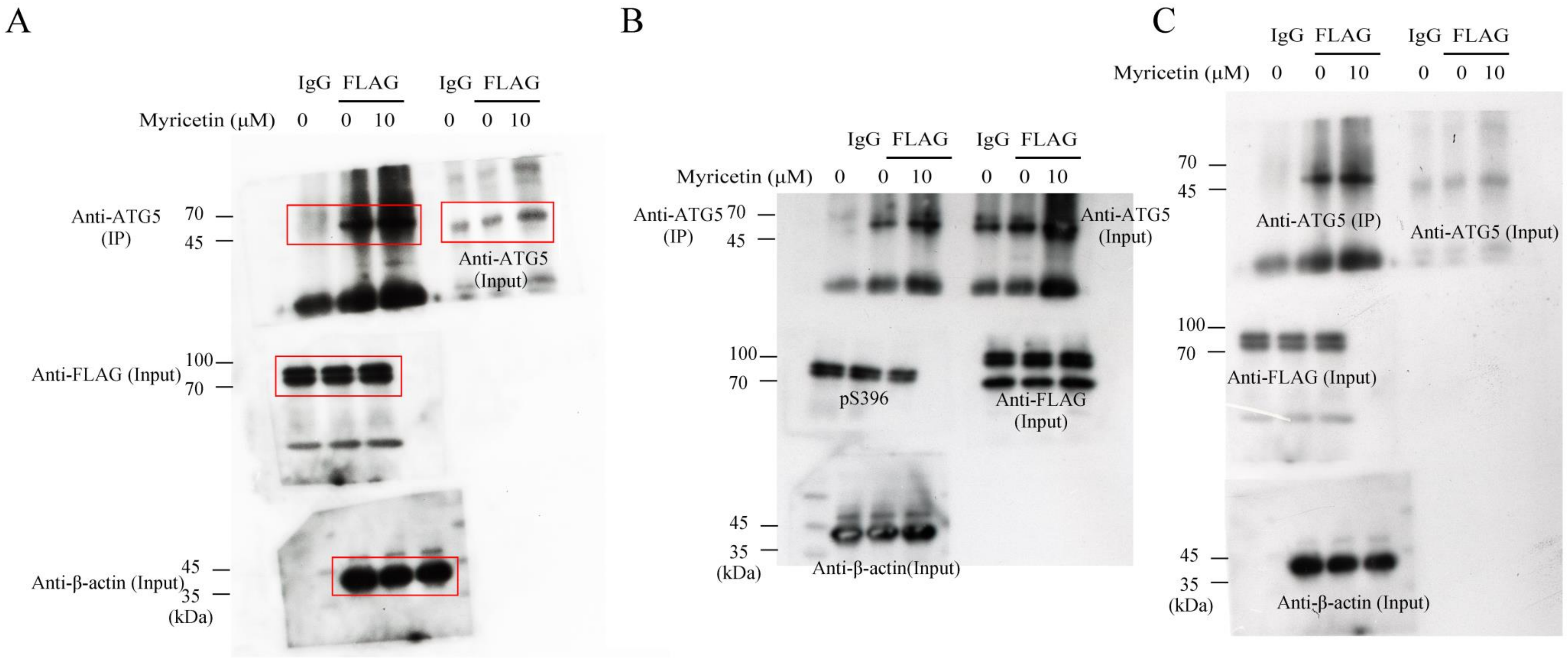
A
IgG FLAG IgG FLAG
Myricetin (μM) 0 0 10 0 0 10
Anti-ATG5 (IP)
70
45
Anti-ATG5 (Input)
Anti-FLAG (Input)
100
70
Anti-β-actin (Input)
45
35
(kDa)
B
IgG FLAG IgG FLAG
Myricetin (μM) 0 0 10 0 0 10
Anti-ATG5 (IP)
70
45
Anti-ATG5 (Input)
100
70
pS396
Anti-FLAG (Input)
45
35
(kDa)
Anti-β-actin(Input)
C
IgG FLAG IgG FLAG
Myricetin (μM) 0 0 10 0 0 10
70
45
Anti-ATG5 (IP)
Anti-ATG5 (Input)
100
70
Anti-FLAG (Input)
45
35
(kDa)
Anti-β-actin (Input)

Figure S7

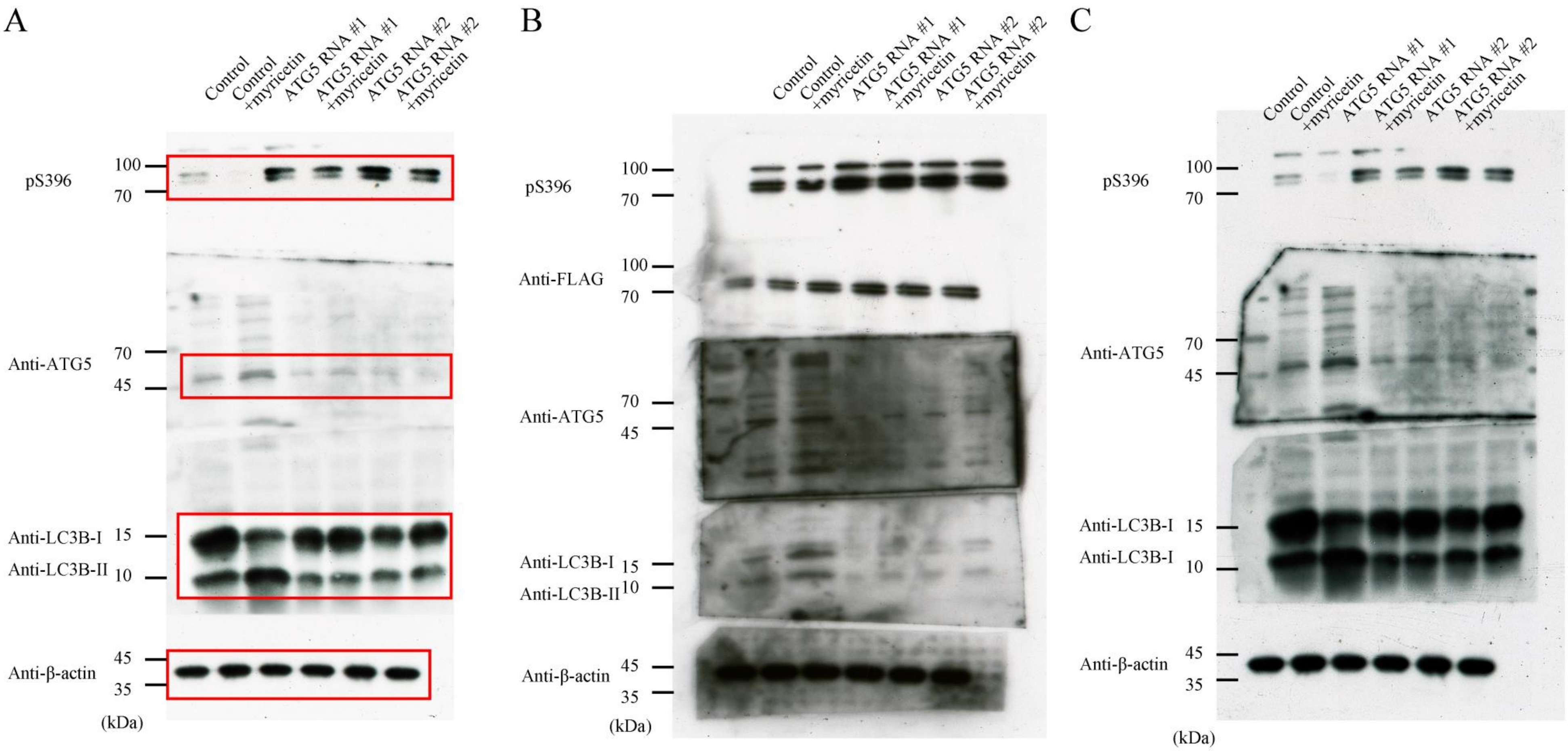
A
Control
Control +myricetin
ATG5 RNA #1
ATG5 RNA #1 +myricetin
ATG5 RNA #2
ATG5 RNA #2 +myricetin
pS396
100
70
Anti-ATG5
70
45
Anti-LC3B-I
15
Anti-LC3B-II
10
Anti-β-actin
45
35
(kDa)
B
Control
Control +myricetin
ATG5 RNA #1
ATG5 RNA #1 +myricetin
ATG5 RNA #2
ATG5 RNA #2 +myricetin
pS396
100
70
Anti-FLAG
100
70
Anti-ATG5
70
45
Anti-LC3B-I
15
Anti-LC3B-II
10
Anti-β-actin
45
35
(kDa)
C
Control
Control +myricetin
ATG5 RNA #1
ATG5 RNA #1 +myricetin
ATG5 RNA #2
ATG5 RNA #2 +myricetin
pS396
100
70
Anti-ATG5
70
45
Anti-LC3B-I
15
Anti-LC3B-I
10
Anti-β-actin
45
35
(kDa)
